# Structure, and Phase Transformations of HPM-3: A Layered Precursor of the Neutral Aluminophosphate with **JSN** Topology

**DOI:** 10.1021/acs.inorgchem.4c04881

**Published:** 2024-12-09

**Authors:** Huajian Yu, Eun Jeong Kim, Zihao Rei Gao, Jeong Hwan Lee, Salvador R. G. Balestra, Chao Ma, Jian Li, Carlos Márquez-Álvarez, Bernd Marler, Suk Bong Hong, Miguel A. Camblor

**Affiliations:** 1 Instituto de Ciencia de Materiales de Madrid (ICMM), CSIC, Madrid 28049, Spain; 2 Center for Ordered Nanoporous Materials Synthesis, Division of Environmental Science and Engineering, POSTECH, Pohang 37673, Korea; 3 Departamento de Física Atómica, Molecular y Nuclear, Área de Física Teórica, Universidad de Sevilla, Seville 41012, Spain; 4 State Key Laboratory of Coordination Chemistry, School of Chemistry and Chemical Engineering, Nanjing University, Nanjing, Jiangsu 210023, China; 5 Instituto de Catálisis y Petroleoquímica (ICP), CSIC, Madrid 28049, Spain; 6 Institute für Geologie, Mineralogie und Geophysik, Ruhr-Universität Bochum, Bochum 44780, Germany

## Abstract

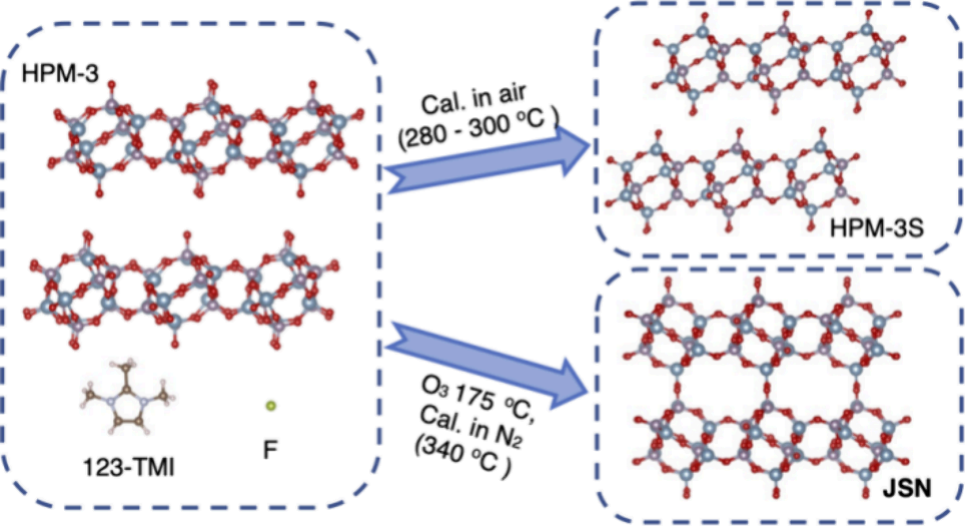

The structure of HPM-3, a layered aluminophosphate prepared using 1,2,3-trimethylimidazolium (123TMI) as an organic structure-directing agent by the fluoride route, has been solved by continuous rotation electron diffraction (cRED), and Rietveld refined against synchrotron powder X-ray diffraction data. Charge balance of the occluded cation is achieved through F^–^ anions and dangling Al(OP)_3_OH groups. Half of the Al is pentacoordinated in negatively charged Al(OP)_4_–F–Al(OP)_4_ pairs. The layers in HPM-3, denoted as *jsn*, are observed in the fully connected metalloaluminophosphate molecular sieves with **JSN** topology. Thus, HPM-3 can be a precursor to a **JSN** metal-free aluminophosphate if a topotactic condensation can be reached, which proved to be difficult but feasible. Treatments under different conditions resulted in a number of known (PST-27 and AlPO_4_-5 by a disruptive transformation) or new phases (through mild thermal treatments). Among the latter, a layered phase with a shorter interlayer space, denoted as HPM-3S, contains half the amount of organic as HPM-3. This is afforded by the capability of 123TMI compounds to sublimate at relatively low temperatures. HPM-3S still contains the same *jsn* layers. Therefore, the transformation of HPM-3 into HPM-3S is topotactic but without reaching condensation. Among the phases appearing under calcination conditions at relatively low temperature (<350 °C), we observed solids compatible with the **JSN** topology, but with poor crystallinity. This is attributed to a wrong alignment in HPM-3 of the dangling Al(OP)_3_OH and P(OAl)_3_=O that should connect adjacent layers in a topotactic condensation, which favors 1-dimensional stacking disorder by random shifts of layers during the thermal treatments. However, detemplation at a much lower temperature (175 °C) using O_3_ finally afforded an aluminophosphate molecular sieve with **JSN** topology with only moderate stacking disorder. This is just the third reported 2D-to-3D topotactic condensations in aluminophosphates.

## Abstract 2

The structure of HPM-3 has been solved by continuous rotation electron diffraction (cRED) and Rietveld refined against synchrotron powder X-ray diffraction data. Charge balance of the occluded cation is achieved through F^−^ anions and dangling Al(OP)_3_OH groups.

## Introduction

The quest to discover new zeolite structures is strongly driven not only by the curiosity, scientific challenge, and beauty of zeolite structures, but also by their proven usefulness in developing/improving specific applications, whether based on catalysis, adsorption, separation, or other types of processes. In the search for new zeolite framework types (ZFTs),^1^ trial and error experiments play an important role. However, these experiments need not to be blind, but can be, and usually are, oriented to maximize the chances of success based on prior knowledge of structure-directing effects.^2^ This oriented trial approach was our aim when we used the 1,2,3-trimethylimidazolium (123TMI) cation (carrying one positive charge) as an organic structure-directing agent (OSDA) at aluminophosphate (AlPO_4_) composition.^3^ The 123TMI cation has proven to be very selective for the crystallization of pure-silica **ITW**,^4^ a phase containing 5-rings, forbidden for AlPO_4_ molecular sieves if Al–P alternation is to be maintained,^5^ so the product of the crystallization should be a different phase. Several AlPO_4_ phases were indeed obtained using this OSDA, although they mainly featured known ZTFs like **CHA** and **AFI**. However, at a high gel concentration (low water content in the reaction mixture), we additionally obtained a new phase with a large amount of occluded species, denoted as HPM-3.

We were not previously able to determine the structure of HPM-3 by high-resolution synchrotron powder X-ray diffraction (PXRD) analysis, despite the clearly defined and distinct nature of its PXRD pattern.^3^ Now, we have finally solved the structure of this new phase from continuous rotation electron diffraction (cRED)^6^ data. Here, we present both the structural and the physicochemical and spectroscopic characterization of HPM-3. HPM-3 does not possess a three-dimensional (3D) zeolitic framework but it is a layered material of zeolitic nature. Its constituent layers are present in the metalloaluminophosphates (MAPO) CoAPO-CJ69 and its Zn analog, with **JSN** topology.^7^ Zeolitic materials with low dimensionalities, such as 2D layers and 1D chains, are interesting as starting materials to develop new derived materials through 2D-to-3D^8^ or even 1D-to-3D^9−11^ topotactic condensation reactions or by interlayer^12^ or interchain expansion reactions,^13^ respectively. These layered materials may be directly synthesized (bottom up) or developed by degermanation of certain germanosilicates within the ADOR methodology (top down) to produce new materials.^14^ In the case of HPM-3, directly synthesized, it can transform into a richness of different phases depending on the conditions: PST-27 (monoclinic **AFI**), standard hexagonal **AFI**, and a well-defined layered material with a shorter interlayer spacing, HPM-3S, as well as several transient phases with varied spacings. Only under carefully controlled mild conditions (slow O_3_ detemplation at low temperature, 175 °C) we could also reach a topotactically condensed neutral and crystalline AlPO_4_ material with the **JSN** topology. Although a few topotactic condensation reactions have been reported in aluminophosphate synthesis,^15^ up to the best of our knowledge this is just the third reported case in which a layered phase experiences a 2D-to-3D topotactic condensation to a microporous AlPO_4_.^16,17^

## Experimental Section

### Synthesis

As previously reported,^3^ HPM-3 was discovered using 123TMI and fluoride as structure-directing agents. Briefly, a gel of composition 1.6 123TMIOH: 1.0 Al_2_O_3_: 1.0 P_2_O_5_: 2 HF: 10 H_2_O was heated at 150 °C while tumbling at 60 rpm. Addition of 1 mol SiO_2_ to the above molar composition also afforded the synthesis of a silicoaluminophosphate (SAPO) with the same framework type (SAPO-HPM-3). Further experiments with composition 2.0 123TMIOH: 1.0 Al_2_O_3_: 1.0 P_2_O_5_: 2.0 HF: *w* H_2_O were also performed at 150 °C (*w* = 5 and 10) and 175 °C (*w* = 10). In all cases, HPM-3 was obtained and found to be stable in the crystallization medium (Table S1). At 175 °C, HPM-3 with good crystallinity was obtained after just 2 h.

### Characterization

Conventional PXRD data were collected in the 3–50° 2θ range on a PANalytical X’Pert diffractometer or a Bruker D-8 diffractometer using Cu Kα radiation. Thermogravimetric/differential thermal analyses (TG/DTA) were performed on a TA SDT Q600 thermal analyzer, and field-emission scanning electron microscopy (FE-SEM) images were obtained on a FEI Nova NanoSEM 230 microscope. C, H, N analysis were obtained in a LECO CHNS-932 or in a Vario EL III elemental analyzers. Multinuclear (^27^Al, ^31^P, ^19^F, and ^13^C) magic angle spinning (MAS) NMR spectra were recorded on a Bruker AV 400WB spectrometer using a triple channel probe with 4 mm ZrO_2_ rotors and Kel-F lids spinning at 10 kHz for ^27^Al, ^13^C, and ^31^P and a double channel probe with 2.5 mm ZrO_2_ rotors and Vespel lids spinning at 25 kHz for ^19^F. ^19^F MAS NMR spectra were acquired at a resonance frequency of 376.45 MHz with a π/8 pulse of 60 kHz, a spectral width of 75 kHz, a relaxation delay of 20 s, and an acquisition of 256 scans, and are referenced using Na_2_SiF_6_ as a secondary reference (−152.46 ppm referenced to CFCl_3_ at δ = 0 ppm as a primary reference). ^13^C {^1^H} cross-polarization (CP) MAS NMR spectra were acquired at 100.61 MHz resonance frequency using a CP-MAS sequence, with a ^1^H excitation pulse of 3 μs, a contact time of 3.5 ms, a recycle delay of 4 s, and a spectral width of 35 kHz, using a proton decoupling power of 80 kHz during acquisition. The spectra are referenced to the CH_2_ resonance of adamantane as a secondary reference (29.5 ppm with respect to TMS at δ = 0 ppm as primary reference). ^31^P MAS NMR spectra were obtained at a resonance frequency of 161.97 MHz with a π/2 pulse of 60 kHz, a spectral width of 100 kHz, relaxation delay of 40 s, and an acquisition of 64 scans. ^31^P {^1^H} CPMAS NMR spectra were acquired with a ^1^H excitation pulse of 3 μs, a contact time of 3 ms, a spectral width of 100 kHz, a proton decoupling power of 80 kHz, a relaxation time of 5 s, and an acquisition of 256 scans. The spectra are referenced using (NH_4_)H_2_PO_4_ as a secondary reference (0.81 ppm from H_3_PO_4_ 85% as a primary reference).

Thermal removal of OSDA from as-made HPM-3 under vacuum was monitored in a temperature-programmed desorption experiment using FTIR analysis (TPD-FTIR). The sample was pressed into a self-supporting wafer of ca. 7 mg cm^–2^ thickness, introduced in an all-glass transmission cell provided with ZnSe windows, heated under dynamic vacuum (residual pressure below 10^–4^ hPa) from room temperature to 321 °C at a rate of 100 °C/h, and kept at constant temperature for 48 h. FTIR spectra were recorded periodically in the 4000–650 cm^–1^ wavenumber range, at 4 cm^–1^ resolution, by averaging 128 scans (total collection time ca. 0.5 min per spectrum), using a Thermo Nicolet Nexus 670 FTIR spectrophotometer equipped with an MCT cryodetector. The kinetics of the low temperature O_3_ detemplation of HPM-3 was followed by Attenuated Total Reflection Fourier Transform Infrared Spectroscopy (ATR-FTIR) using a Nicolet Nexus 670 spectrometer provided with a GladiATR single-bounce monolithic diamond ATR accessory and an MCT cryodetector. The spectra were recorded in the 4000–650 cm^–1^ range, at 4 cm^–1^ resolution, by averaging 128 scans.

### O_3_ Treatments

Low-temperature detemplation of HPM-3 was carried out under an ozone-oxygen mixture flow. An Ozonosystem ECO_3_ C-3 electrical discharge ozone generator was used to produce O_3_ from high-purity oxygen (≥99.995 vol %) at a rate of 500 mg h^–1^.

### Structure Solution and Refinement

cRED data were obtained in a JEOL JEM2100 transmission electron microscope (TEM) with a LaB_6_ filament (Cs, 1.0 mm; point resolution, 0.23 nm) operating at an acceleration voltage of 200 kV. During the data collection, the goniometer was rotated continuously, while the selected area ED patterns were captured from the crystal simultaneously by a quad hybrid pixel detector (Timepix, 512 × 512 pixels with a size of 55 μm, Amsterdam Sci. Ins.). High-resolution synchrotron PXRD data of HPM-3 were also collected in capillary mode at the Spanish beamline BM25A (λ = 0.82548 Å, from 3 to 46.55 ^*o*^ 2θ) of the European Synchrotron Research Facility, ESRF, Grenoble (France). Rietveld refinement was performed using those data and the structural model obtained from cRED. Structure images have been produced using the VESTA software.^18^

### Calculations

Energy minimization of the structures was performed using the GULP software.^19^ To accurately capture template-template and framework-template interactions, periodic boundary conditions were applied. A 16 Å cutoff was used for calculating short-range interactions in real space, while the Ewald summation method was employed for long-range interactions. Both the cell parameters and ionic positions were optimized for each configuration, with a convergence criterion set at 0.001 eV Å^–1^ for the forces. Initially, the Newton–Raphson minimizer was applied, which updates the Hessian matrix through the Broyden–Fletcher–Goldfarb–Shanno (BFGS) approximation, followed by the rational function optimization (RFO) or eigenvector-following method. This approach generally ensures convergence to true minima (i.e., with no imaginary vibrational modes), which is particularly relevant for zeolitic materials.^20^ To describe interactions between atoms, we employed the potential developed by Gale and Henson for AlPO_4_ materials.^21^ The calcined AlPO_4_-**JSN** structure was optimized energetically using the structure in the IZA database, with an AlPO_4_ composition. The cif file is available in the Supporting Information.

## Results and Discussion

**Figure 1:**
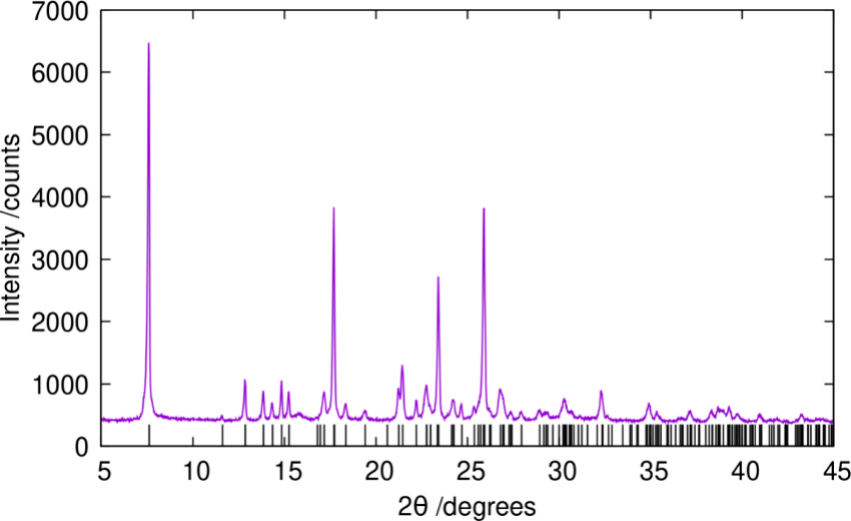
PXRD pattern of HPM-3. The vertical lines mark allowed reflections for the initial indexing results (space group *Cc*, *a* = 23.503 Å, *b* = 7.21 Å, *c* = 15.417 Å, β = 98.90°, λ = 1.54186 Å).

HPM-3 is a highly crystalline solid, and its PXRD pattern was successfully indexed as monoclinic, space group *Cc* or *C*2/*c*, with *a* = 23.503 Å, *b* = 7.21 Å, *c* = 15.417 Å, β = 98.90° (Figure 1). However, our attempts to solve its structure by direct methods and simulated annealing using high resolution synchrotron PXRD data and EXPO13^22^ failed, although the simulated annealing results suggested a layered structure. The program TOPAS^23^ was employed to perform the simulated annealing using two types of rigid bodies, TO_2_ units and the 123TMI cation, and making use of so-called “anti-bump” constrains preventing unfeasible proximity or even overlap of rigid bodies. The erroneous space group symmetry, *Cn*, assumed to be correct at this stage of the analysis possibly prevented a complete structure determination.

**Figure 2:**
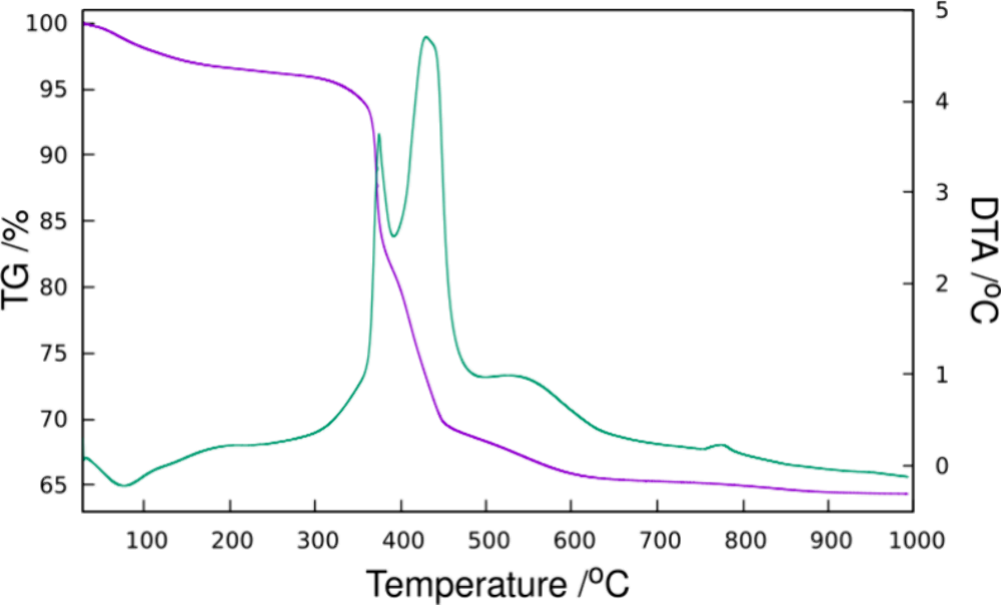
TG (purple) and DTA (green) curves of HPM-3 in air.

Elemental analysis (Table 1) revealed a large organic content (C + H + N = 29.1 wt%) and C/N and H/N ratios close to the values in the pristine 123TMI. This large organic content was confirmed by TG/DTA analysis in air, with a first endothermic loss of around 3.77 wt%, followed by a very large loss composed of at least three exothermic events (maxima around 377, 430, and 540 °C) and a less remarkable exothermic loss around 775 °C (Figure 2). The total weight loss amounts to 35.7%, most of which (ca. 30.5%) was lost below the remarkably low temperature of 450 °C. The first weight loss probably involves H_2_O and partial organic removal (see below). Combining chemical analysis, TG, and crystallographic data (see below), the formula for HPM-3 is proposed in Table 1. We also included data for the related material HPM-3S, where S indicates “short” (see below).

**Table 1:** Chemical Composition of HPM-3 and HPM-3S

**Phase**	**wt %**			**molar ratios**		**wt %**	
	**N**	**C**	**H**	**C/N**	**H/N**	**TG residue**	**unit cell**^a^
HPM-3	7.2	18.9	3.0	3.1	5.8	64.4	[C_6_H_11_N_2_]_16_[AlPO_4_]_32_(OH)_8_F_8_·(H_2_O)_3.2_
expected^b^	7.4	19.1	3.2	3.0	6.0	64.7	
HPM-3S	4.1	10.2	2.4	2.9	8.2	75.7	[C_6_H_11_N_2_]_7.5_[AlPO_4_]_32_(OH,F)_7.5_(H_2_O)_16_
expected^b^	4.1	10.5		3.0	8.2	75.7	

a Based on the crystallographic study and TG and C, H, and N analyses. For HPM-3, the cell formula comes from the crystal solution adding water to account for the TG residue. For HPM-3S, 123TMI derives from N% considering that the residue is AlPO_4_ and it is assumed that Al(O_3_)(OH,F) groups counterbalance the cation (see below). Water is calculated to account for the TG residue.

b Theoretical values for the composition in the last column. The uncertainty about the amount of F replacing OH in HPM-3S (see below) hinders the calculation of H %.

### Phase transformation to **AFI**

Upon calcination in air, as-synthesized HPM-3 transformed into AlPO_4_-5 (**AFI**) with a 1D 12-ring channel system, and the transformation took place in the temperature range (Figure S1) in which most organics were removed (Figure 2). Interestingly, it was found that digestion in plain water at 180 °C for 3 h also transformed HPM-3 into **AFI**, whereas the resulting **AFI** phase had a monoclinic structure, much similar to PST-27,^3^ suggesting the inclusion of OSDAs in its pores (Figure S2). The same transformation occurred at pH = 2.5 (reached by adding acetic acid). At pH = 10.5 (reached by adding ammonia), however, AlPO_4_–CJ2 (with an impurity) was obtained.^24,25^ We also note that digestion of SAPO-HPM-3 in plain water gave **AFI**, whose PXRD was closer to the standard hexagonal **AFI** phase than to PST-27. OSDA inclusion in the digested solid with monoclinic PST-27 PXRD pattern was supported by TG/DTA, showing an exothermic weight loss of 15% at relatively high temperatures (350–600 °C, maximum in DTA at 470 °C) that was preceded by an endothermic weight loss of 1.6% below 250 °C (Figure S3).

The transformation of HPM-3 into **AFI** by calcination or digestion in plain water is reminiscent of JDF-20, an interrupted AlPO_4_ phase with 20-ring pores synthesized in a predominantly nonaqueous medium using triethylamine without fluoride.^26,27^ JDF-20 was also reported to transform into **AFI** by heating in air above 300 °C or by digestion in water at 180 °C. JDF-20, however, has an excess of P over Al, unlike the case of HPM-3, and its TG weight loss is much smaller (25–29 wt %).^27^

An *in situ* high-temperature PXRD study in air (Figure S1) shows that HPM-3 has already transformed into **AFI** at 400 °C (some tridymite-like AlPO_4_ was also formed in these conditions). The very large organic content, low combustion temperature, and easy transformation to **AFI** suggested that HPM-3 might not be a fully connected framework material but, rather, it might either possess a lower dimensionality and/or be an interrupted framework.

HPM-3 crystals are relatively small and irregular in shape and appear to be composed of smaller and irregular log-shaped crystals. After calcination, better faceted, smaller and more regular prisms, roughly reminiscent of hexagonal prisms, developed (Figure S4). The large change in morphology supports the idea that the phase transition involves a large structural transformation. As we will show below, HPM-3 is a layered material (framework density, FD = 12.4 T/1000 Å^3^) while AFI is a much denser framework zeolite (FD = 16.9).

### Multinuclear MAS NMR

**Figure 3:**
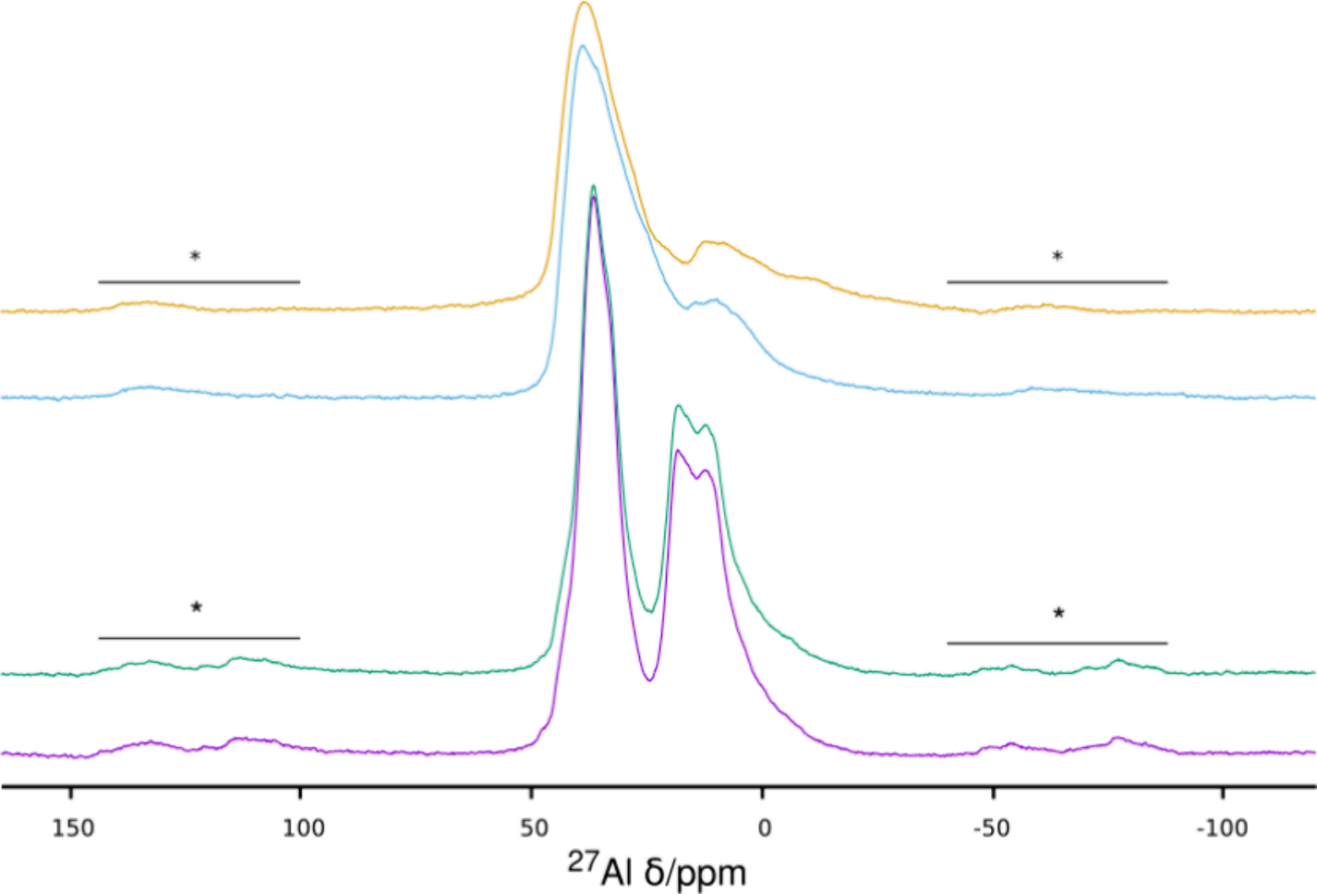
^27^Al MAS NMR spectra of (from bottom to top): dried and undried HPM-3 and dried and undried HPM-3S. The regions with spinning side bands are marked with lines and asterisks.

**Figure 4:**
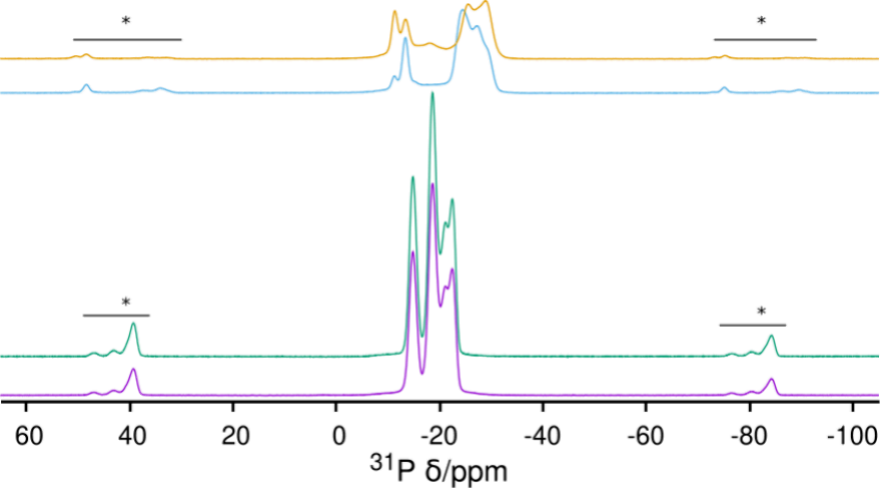
^1^H–^31^P CP MAS NMR spectra of (from bottom to top) dried and undried HPM-3 and dried and undried HPM-3S. Regions of spinning side bands are marked with lines and asterisks.

We collected the ^27^Al MAS NMR spectra of HPM-3 in dried (100 °C overnight) and undried states (Figure 3), because the framework Al atoms in AlPO_4_ molecular sieves could change coordination in the presence of water. Al was found to adopt tetra- (35 ppm) and pentacoordination (18 and 12 ppm) with little changes related to the hydration state. The ^31^P CPMAS NMR spectrum (Figure 4) was not very informative, due to the high sensitivity of ^31^P chemical shifts to the average P–O–Al angles, which is also the case even when P–O_t_ bonds (terminal O as in P=O or P–OH) exist.^28^ All four resonances at −22.4, −21.0, −18.6, and −14.8 ppm can be assigned to tetrahedral P atoms (there are 8 inequivalent P-sites in HPM-3, see below),^29^ and no changes were observed related to the hydration state. We also note that while the direct irradiation spectrum shows the same resonances with a poorer resolution (Figure S5), two small additional resonances at −2.0 and −11.8 ppm appeared after rehydration, which might perhaps indicate the presence of some impurity in that particular sample.

**Figure 5:**
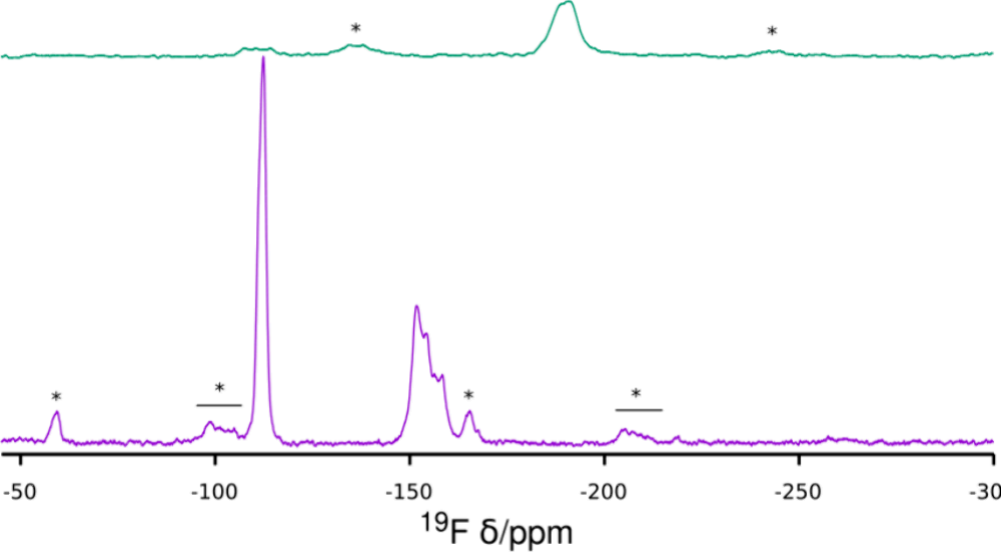
^19^F MAS NMR spectrum of HPM-3 (bottom) and HPM-3S (top). Regions of spinning side bands are marked with lines and asterisks.

The ^19^F MAS NMR spectrum of HPM-3 (Figure 5) shows a strong, sharp and symmetric resonance at −112.3 ppm, assignable to F bridging to pentacoordinated Al (i.e., Al(OP)_4_F(OP)_4_Al; see below). A similar chemical shift value (−115 ppm) has been observed for Al–F–Al bridges in a detailed NMR study of zeolitic AlPO4-CJ2.^30^ There are also several overlapped resonances in the range −150 to −160 ppm. Since the range of these resonances is so far away from the one commented above and, according to the structure analysis (see below), there are only two crystallographically different fluorides in highly similar environments, we speculate that the high field signals might correspond to impurities in HPM-3 not observable by PXRD^30^ (or, alternatively, to terminal Al–F units partly replacing terminal Al–OH units, see below). The OSDA 123TMI is occluded intact in HPM-3, according to ^13^C CPMAS NMR (Figure S6).

### Structure Solution of HPM-3

**Figure 6:**
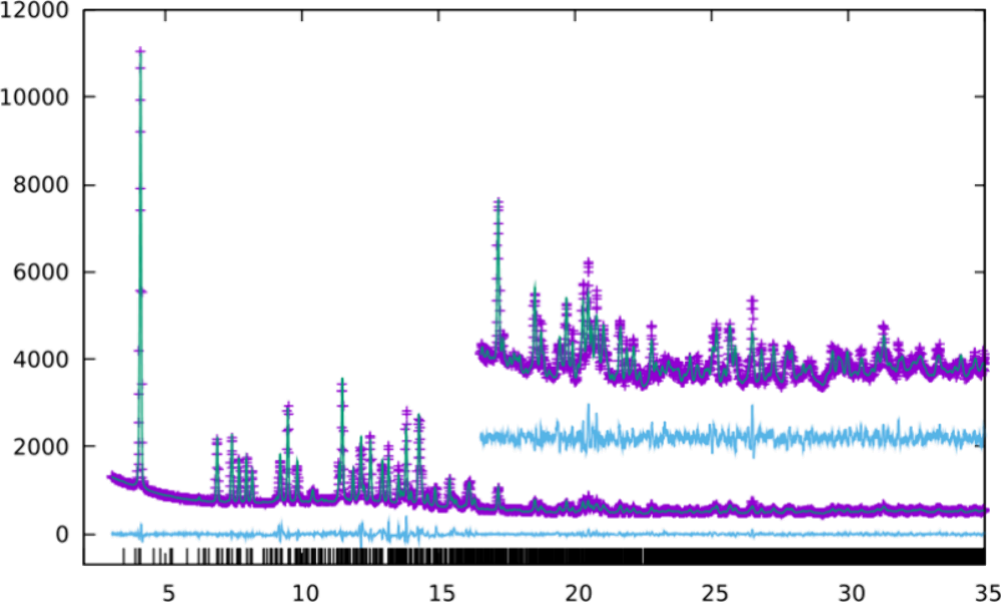
Observed (purple crosses) and calculated (green solid line) PXRD patterns for as-synthesized HPM-3 refined in space group *P*2_1_/*n*. Vertical marks indicate the positions of allowed reflections. The lower cyan trace is the difference plot. λ = 0.82548 Å.

We were able to solve the HPM-3 structure using cRED data: eight data sets were collected on individual crystals of HPM-3 (Table S2; the data for HPM-3S are shown in Table S3). The reciprocal space reconstruction (Figure S7) was done using the program REDp,^31^ and the reflection intensity extraction was conducted with the program XDS.^32^ The data were scaled and merged based on unit cell consistency and correlation coefficients between the data sets using XSCALE in the XDS suit. Indexing revealed a doubling of the cell and a symmetry lowering to *P*2_1_/*n*, in contrast to the initial indexing from PXRD data. The structure solution clarified this issue, since Al–O–Al and P–O–P bonds would exist in the original indexing. With the high data resolution of 0.81 Å and completeness of 98.1%, *ab initio* structure solution was performed using SHELXT,^33^ which directly located all non-H atoms. Then, the structure model was refined by Olex2^34^ with atomic scattering factors for electrons. Crystallographic details are presented in Table S4. In order to obtain more precise bond lengths and angles, we refined the structure of HPM-3 against synchrotron PXRD data by the Rietveld method using GSAS-II.^35^ The starting model for the refinement was the structure solution obtained by cRED. The Chebyschev-1 function with 12 coefficients was settled and refined to fit the background during the refinement. A pseudo-Voigt function was used for peak-shape fitting before the structure refinement. Initially, soft restraints on P–O (1.53 Å), Al–O (1.80 Å), O–P–O (109.54°), O–Al–O (109.54° in tetrahedral Al, 120° and 90° in trigonal bipyramid Al) and F–Al–O (90°) were used in the refinement, and the restraints weight factors were reduced gradually up to a final weight of 0.1 in GSAS. The OSDA was treated as a rigid body and allowed to optimize position, rotation angle, orientation vector and displacement parameters. The Rietveld plot is shown in Figure 6, and the crystallographic data and structural details are shown in Tables S5–S7. A cif file is provided as Supporting Information.

**Figure 7:**
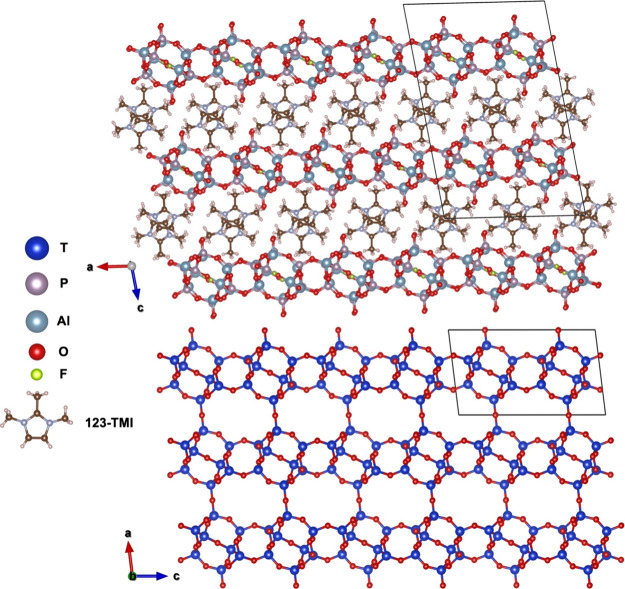
Rietveld refined layered structure of HPM-3 (top), showing unconnected *jsn* layers identical to those in the MAPO materials with **JSN** topology (bottom). Unit cell borders are drawn with thin black lines.

The structure solution indicates that HPM-3 is indeed a layered material and contains a large amount of 123TMI cations (Figure 7). Interestingly, the layers in HPM-3, designated *jsn* here, are also observed in the fully connected **JSN** zeolite topology.^1^ It is interesting to note that only the metalloaluminophosphates (MAPO) molecular sieves CoAPO-CJ69 and its Zn analog,^7^ but no neutral AlPO_4_ counterpart, possess the **JSN** topology. These MAPO-CJ69 were synthesized in a mixture of tetraethylene glycol and water using the small diethylamine as an OSDA, with no fluoride present.^7^

**Figure 8:**
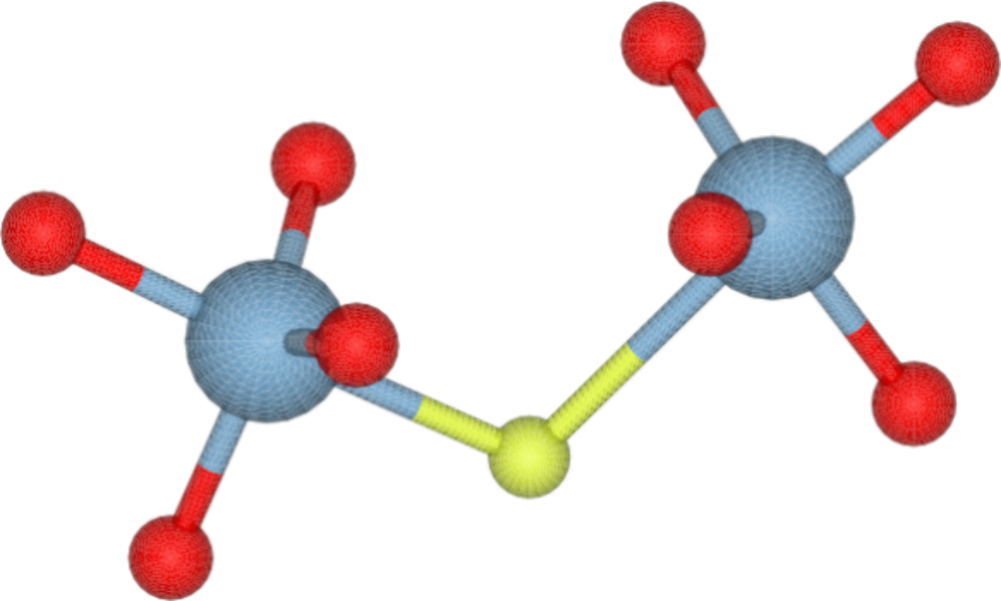
A pair of pentacoordinated Al atoms in HPM-3, sharing a fluoride anion and displaying a regular trigonal bipyramid geometry with a long apical Al–F bond. The color code is the same as that in Figure 7.

HPM-3 contains 32 Al atoms per unit cell, and eight different Al atoms in the asymmetric unit. Half of them are pentacoordinated AlO_4_F units, sharing eight fluoride anions in pairs (Figure 8). It should be noted that while half of these pentacoordinated AlO_4_F units are bonded to four PO_4_ tetrahedra, the other half have only three P neighbors and a dangling bond to oxygen that we assume to be an Al–OH group and that faces the interlayer space. Thus, charge balance of the 16 123TMI cations per unit cell is achieved through 8 [AlO_4_]F[AlO_4_] pairs and 8 Al(OP)_3_(OH) groups, both types of groups bearing a negative charge. In contrast, pentacoordinated Al is completely absent in MAPO-CJ69.^7^ There are also 32 P atoms per unit cell in HPM-3, eight per asymmetric unit. All P atoms are tetracoordinated. However, one-quarter of them have P = O terminals facing the interlayer space.

The ideal unit cell formula of as-synthesized HPM-3 derived from the structure solution is thus |C_6_H_11_N_2_|_16_[AlPO_4_]_32_(OH)_8_F_8_. Protons have been added in order to have a neutral structure and are part of the dangling Al–OH groups mentioned above. No hexacoordinated Al was found by cRED. A GULP optimization of the structure with fluoride provided results much coincident with the crystallographic results.

### Short-Edge HPM-3 (HPM-3S)

Given that the *jsn* layers in HPM-3 are also present in **JSN** MAPO materials, we tested the possibility of a topotactic condensation from HPM-3 to **JSN**. Topotactic condensation from HPM-3 should result in a neutral AlPO_4_ material with **JSN** topology, which had never been achieved before. Given that a direct calcination at 550 °C resulted in the formation of **AFI** (Figure S1), we first investigated the *ex situ* calcination of HPM-3 under different conditions in air (Table S8).

Deep bed calcination at 450 °C or higher resulted in the formation of AlPO_4_-5 (**AFI**), as mentioned above. However, under shallow bed conditions, calcination led to structural collapse (amorphization, partial at 450 °C and total at higher temperatures). We also performed experiments with water vapor to simulate a more humid ambient (marked as SARC in Table S8). For this, we placed HPM-3 in a small open container placed inside a Teflon liner of an autoclave, and some water between the container and the Teflon liner. The product of these transformation experiments was found to depend on the HPM-3/water ratio: when the amount was relatively large, after heating to 190 or 200 °C the final solid was found soaked in liquid and the product was PST-27, similarly to the hydrothermal experiments at 180 °C described above. When the amount of water was much smaller no phase transformation occurred at 190 °C.

**Figure 9:**
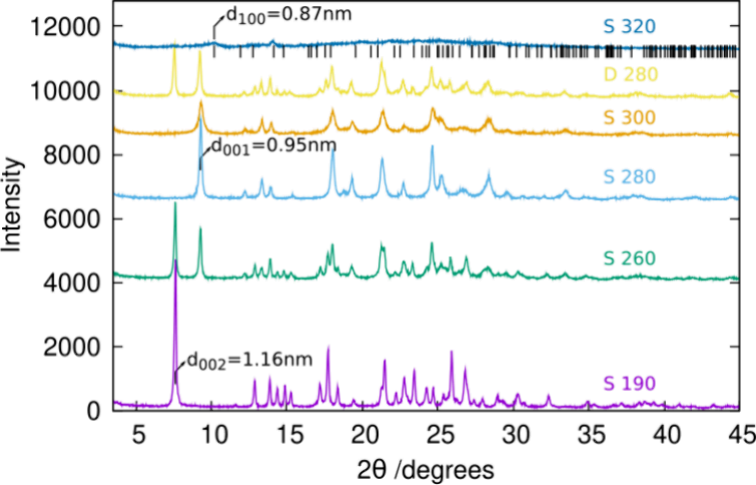
Low-temperature treatment of HPM-3 in air. The colored labels indicate the temperature of each treatment while S and D stand for ″shallow-bed″ and ″deep-bed″ calcination, respectively. Values of *d*-spacings related to the interlayer distances for HPM-3, HPM-3S and **JSN** are marked. Close to the upper pattern, allowed reflections for **JSN** are marked with vertical lines.

The most interesting results were found when HPM-3 was treated at low temperatures in air (Table S8 and Figure 9). Under shallow-bed conditions, no transformation occurred at 190 °C, but at 260 °C a new well-defined phase started to emerge and became a single phase at 280–300 °C. This phase was characterized by one shorter cell edge that, after structure elucidation by cRED (see below), was found to correspond to the interlayer spacing. In other words, the observed phase change is a topotactic transition that shortens the interlayer space, but results in no condensation of the layers. We denote this phase HPM-3S. HPM-3S contains half the amount of organics as the parent HPM-3 (Table 1). Further heating at 320 °C resulted in significant structural collapse, although there are small and broad reflections around 10.3, 11.8, and 14.1° that are coincident with those expected for **JSN**. This suggests that the topotactic condensation might have taken place to some extent but resulted in a very low-crystallinity material (see below). Deep-bed calcination at 280 °C produced a mixture of both the long and short HPM-3 versions.

The results described above can be explained as follows: deep-bed calcination at high temperatures produces the sudden combustion of a very large amount of OSDA occluded in the interlayer space of HPM-3, likely producing much higher local temperatures and water. If such is the case, then the extra energy and water would promote the transformation into **AFI**. Under shallow-bed conditions, on the other hand, heat can be dissipated more rapidly, hindering the transformation. Under conditions that would supposedly afford a steam assisted conversion, on the other hand, transformation into PST-27 occurs when water condenses in the vessel containing HPM-3, effectively converting the treatment into a hydrothermal process. We note that, as in any other change of phase, provided there is enough water there must be a dynamic equilibrium between liquid and vapor water. This may convert steam-assisted conditions into purely hydrothermal ones, depending on the formation of liquid water and thus on the amount of water added and the volume of the autoclave. Finally, at temperatures that are clearly below the sudden combustion of OSDAs in the dynamic TG experiment (Figure 2), half of the 123TMI cations can be removed from HPM-3, resulting in a phase with a shorter interlayer space (i.e., HPM-3S). This conclusion can be supported by elemental and thermal analyses on HPM-3S (Table 1 and Figure S8). The remaining 123TMI is still intact (Figure S6).

The TG/DTA data of HPM-3S (Figure S8) were found to show two endothermic weight losses at low temperature: one (about 3 wt %) up to 100 °C and the other (ca. 1.5 wt %) up to 180 °C. Since these processes occurred at temperatures well below the sublimation of 123TMI (see below), we assign them to water molecules in two different states, i.e., molecules physisorbed and chemically bonded to framework Al (see below), respectively. On the other hand, the high-temperature weight loss of approximately 20 wt % starts around 400 °C and is associated with two exothermic processes. Given their temperatures, we mainly assign them to OSDA desorption and combustion. However, considering the unit cell formula in Table 1, the amount of water (16 per unit cell, or 5.6 wt %) exceeds the amount of weight lost in the low temperature step (4.5%). This suggests that the high temperature loss (19.8 wt %) does not correspond only to 123TMI and water coming from dehydroxylation of Al(OP)_3_OH groups (which would amount to 17.5 wt % at most) but should also involve other species (for instance water coordinated to Al, see ^27^Al MAS NMR below).

The ^27^Al MAS NMR spectra of HPM-3S (Figure 3) reveal that the coordination number of Al is still mainly four and five, although some hexacoordinated Al exists. The intensity of the latter Al decreases after drying at 100 °C overnight. On the other hand, the ^31^P MAS NMR spectra of HPM-3S taken with (Figure 4) or without CP (Figure S5) show that the resonances now span a much broader chemical shift range from −10 to −30 ppm and this happens because all the resonances are shifted either high or low field compared to the previous chemical shift range (−14 to −23 ppm), except for a resonance at −18 ppm that merges with the background after drying.

The ^19^F MAS NMR spectrum of HPM-3S shows that fluoride is partly retained in the solid (Figure 5). Energy dispersive spectroscopy results suggest that the Al/F ratio decreases from 0.5 in HPM-3 to 0.3 in HPM-3S. However, besides a small broad resonance at a chemical shift of −111 ppm, similar to that observed in HPM-3, most fluoride changed drastically its chemical shift to −190 ppm. This very high field ^19^F resonance implies a large change in the fluoride environment to a more screened one and might imply the formation of fluorophosphates, as observed in fluorophosphate glasses, where the ^27^Al NMR remains largely unaffected.^200−37^ However, considering charge balance, ^19^F MAS NMR and the existence of charged terminal Al, pentacoordinated Al and water heavily bound to the framework we propose the partial conversion of charged Al(OP)_3_(OH) into charged Al(OP)_3_(F) and the existence of uncharged [AlO_4_](H_2_O) pentacoordinate units (after exposure to ambient air). It is unlikely that the bare 123TMI cation could sublimate, whereas a significant portion of fluoride is retained in the solid. Therefore, the proposed (partial) substitution of OH by F in terminal Al groups could afford evolution of TMI in the form of TMIF and TMIOH (or the products of the cation degradation catalyzed by OH^–^).

After the HPM-3 to HPM-3S topotactic transition the retained organic imidazolium remains intact, as evidenced by ^13^C CPMAS NMR, which just shows some small variations in chemical shift (Figure S6). The most shifted resonance corresponds to the C atom between both N in the imidazolium ring that changes from 146 ppm in HPM-3 to 144 ppm in HPM-3S. The other aromatic C atoms (the symmetrical C4 and C5) resonances shift slightly from 122 to 123 ppm and become significantly broader. A general broadening of ^27^Al, ^31^P, ^19^F and ^13^C NMR resonances (Figures 3–5 and Figure S6) and of PXRD peaks (Figure 9) suggests that the degree of structural order is slightly lower in HPM-3S than in HPM-3.

The structure of HPM-3S was solved using cRED data, as was the case for the long version. For this, six data sets were collected on individual crystals of HPM-3S (Table S3). Data process and structure solution were performed as with the long version and showed that HPM-3S maintains the layered structure of HPM-3, but with a shorter interlayer distance between *jsn* layers. The crystallographic data are presented in Table S4. Unfortunately, we were not able to locate water, fluoride and 123TMI ions from cRED data, which is likely due to the poor orderliness of the guests in that phase after the heating treatment. The partial structure of HPM-3S is compared to that of HPM-3 in Figure S9.

### Heating under N_2_

Given the results presented so far, we initially attributed the failed or deficient topotactic condensation of HPM-3 to an AlPO_4_ material with **JSN** topology to the extreme local energies and water produced by OSDA combustion in air. Therefore, we decided to try calcination under inert atmosphere as well. To our surprise, the TG and DTA results in air and in N_2_ or Ar revealed that not only the weight lost at each step but also their temperature range and the total weight loss are very similar in the three experiments (Figure 2, Figures S10 and S11). The solid residues after TG/DTA under the three atmospheres were all white and their PXRD patterns showed a transformation to **AFI** (with varying proportions of tridymite-like AlPO_4_). A TG analysis under Ar showed that the iodide salt of 123TMI gets rapidly volatilized with a 100% weight loss starting at 315 °C and ending at 360 °C (Figure S10). This sublimation has to be compared with the weight loss of HPM-3 which starts at 360 °C under air and 370 °C under both N_2_ and Ar, all of them ending around 500 °C. As previously discussed, and given that a significant portion of F is retained in HPM-3S (see above), we propose that a fraction of hydroxide anions in the Al–OH dangling groups is replaced by fluoride, so that the species sublimating from HPM-3 might be TMIOH and TMIF, perhaps explaining the two steps in the TG curves in Figure S10). Since all these thermal analyses are dynamic experiments we tried to get further insights into the kinetics of the sublimation by a thermal treatment in vacuum followed by FTIR. The results show that at a fixed temperature of 321 °C in vacuum there is a very slow and prolonged release of organics (Figure S12).

As commented above, our attempts to achieve a topotactic condensation by calcination in N_2_ at different temperatures and for different durations, were aimed to avoid excessive local heating. As seen in Table S9, most experiments produced either **AFI** (dominant above 400 °C) or HPM-3S, but some treatments at low temperature and for a long time resulted in a phase with a very low crystallinity that could possibly be a disordered **JSN** (Figure S13). This phase is very similar to the one observed by shallow bed calcination at 320 °C in air (Figure 9, top pattern).

### Effective Topotactic Condensation to AlPO_4_-**JSN**

Finally, since we have recently proven that ozonolysis is a convenient way to ″detemplate″ imidazolium-loaded zeolites at low temperatures (below 150 °C),^36^ we tried ozonization of HPM-3. In contrast to the observed behavior in large and extralarge pore zeolites, which can be detemplated at 100 °C, detemplation of HPM-3 proved somewhat difficult and after treatment at 150 °C for 35 h it was far from complete (Figure S14). After such a treatment the sample is a mixture of HPM-3 and still another phase (Figure S15, bottom): this new phase has its first reflection peak at around 8.7°, an angle intermediate between the low angle peaks of HPM-3 (9.2°) and HPM-3S (7.6°). Calcination of that phase in N_2_ at 250 °C increases its crystallinity and decreases the proportion of HPM-3 (Figure S15). Further calcinations in N_2_ show that it is possible to convert all HPM-3 while decreasing the interlayer space even beyond that of HPM-3S but without reaching the value expected for the **JSN** structure (Table S9). All these ″transient″ phases have a lower crystallinity than HPM-3 and HPM-3S.

**Figure 10:**
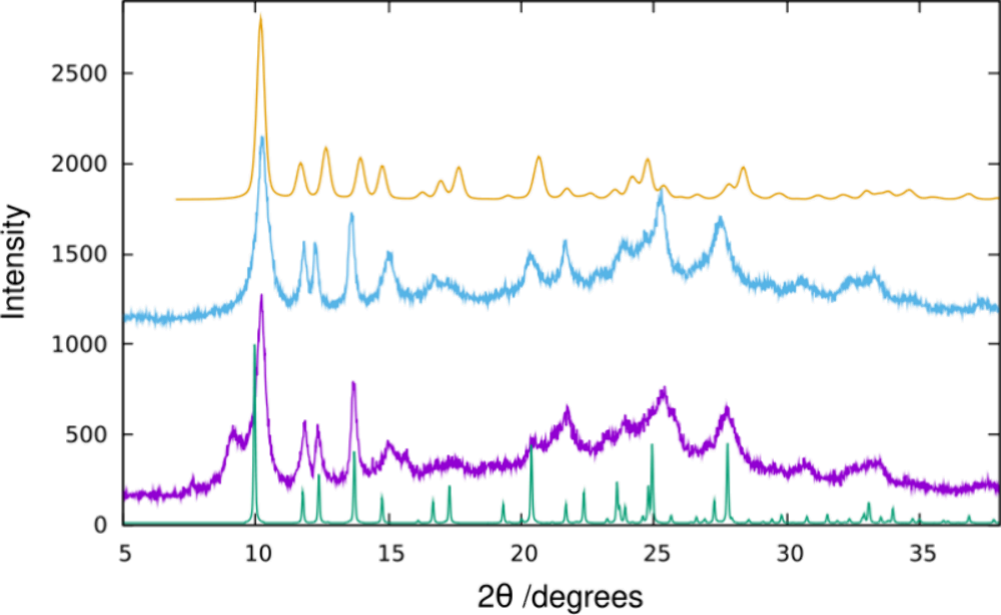
PXRD pattern of (from bottom to top): a simulation of the CoAPO-CJ69 with **JSN** topology obtained from the IZA database (green),^1^ HPM-3 after O_3_ treatments at 175 °C for 24 h, (purple, Figure S16), the same followed by calcination in N_2_ at 340 °C (6 h ramp, 48 h plateau, cyan) and a simulation of the PXRD of AlPO_4_-**JSN** optimized using Gale and Henson’s potential with the GULP code.^19,21^

A harsher O_3_ treatment of HPM-3 at increasing temperatures revealed that, even after 9 h at 175 °C, some organics remained. However, the treatment at that temperature for 24 h left no organics detectable by ATR (Figure S16). The resultant solid appeared to be mainly **JSN** with a small amount of HPM-3S remaining, which disappeared after calcination at 340 °C in N_2_ (Figure 10). The PXRD of the final solid resembles very much the pattern of **JSN**-type CoAPO-CJ69 obtained from the Database of Zeolite Structures, with some differences likely related to the different unit cell dimensions (Figure 10). Furthermore, a PXRD simulation of a GULP-optimized neutral AlPO_4_-**JSN**, Figure 10, fully confirms the conclusion that the topotactic condensation has been reached.

The PXRD of this AlPO_4_-**JSN** solid shows very broad reflections and a rapid decrease of the reflection intensity with increasing diffraction angle. Both observations are indicative of a certain degree of structural disorder. Still, sufficiently resolved reflections are present which can be indexed based on a *P*2_1_/*c* monoclinic unit cell with lattice parameters *a*_0_ = 8.691 Å, *b*_0_ = 14.491 Å, *c*_0_ = 15.053 Å and β = 94.344°. The resolved reflections allowed to perform a Rietveld refinement of the **JSN**-AlPO_4_ structure. The slightly modified structure of CoAPO-CJ69 served as the starting model (Co atoms were replaced by Al atoms and the eight nitrogen and carbon sites of diethylamine molecules being present in CoAPO-CJ69 were replaced by three water sites in **JSN**-AlPO_4_). Because of the limited information available, the atomic coordinates were refined with relatively strict distance restraints (*d*_P–O_ = 1.54(1) Å, *d*_Al–O_ = 1.72(1) Å, *d*_O···O_ = 2.65(2) Å, *d*_P···Al_ = 3.10(1) Å) and meaningful displacement parameters were kept fixed: *B*_iso_(P) = 1.6 Å^2^, *B*_iso_(Al) = 1.6 Å^2^, *B*_iso_(O, framework) = 3.2 Å^2^, and *B*_iso_(O, water) = 5.0 Å^2^. Six additional halfwidth parameters were used to account for anisotropic peak broadening provoked by the disorder.

The Rietveld refinement based on the PXRD data available did not yield a fully satisfying result (chi^2^ = 2.66, *R*(p) = 19.8, *R*(F) = 6.39). Nevertheless, the fact that the refinement of the AlPO_4_-**JSN** structure led to a fair fit of experimental and calculated powder patterns (see Figure S17) with refined values of bond distances and angles in a meaningful range (*d*_Al–O_ = 1.54–1.61 Å, *d*_P–O_ = 1.65–1.72 Å, *d*_P···Al_ = 3.04–3.18 Å, *d*_O···O_ = 2.50–2.86 Å) clearly proves that the O_3_ treated sample, on average, has the **JSN** structure, with an only moderate degree of disorder

Careful examination of the HPM-3 structure shows that the dangling Al(OP)_3_OH and P(OAl)_3_=O groups, which face the interlayer space and should afford the connection of the layers, do not appear to be aligned in a way that is conductive to an easy connection by topotactic condensation. Rather than terminal Al and P being aligned they are interspersed in adjacent layers when viewed along *a* (Figure S18). In fact, the distance between terminal P atoms in adjacent layers (7.20 Å) is smaller than the distance between terminal Al and P atoms (7.44–7.76 Å). A similar situation is observed in HPM-3S because, although the interlayer space is shortened, there is a lateral displacement of the layers along *a* (Figure S9). Thus, the terminal P–P distances between layers (6.9–7.2 Å) are still shorter than terminal P–Al distances (7.2–8.4 Å). This misalignment, and random shifts of layers perpendicular to the stacking direction occurring during the heating, would make it difficult to result in a straightforward topotactic condensation, decreasing long-range order and resulting in 1D stacking disorder. However, detemplation by ozonization at a significantly lower temperature (175 °C) reduces this disordering effect and affords the neutral AlPO_4_ material with **JSN** topology and an improved crystallinity.

We can now consider the causes for the scarce occurrence of 2D-to-3D topotactic condensations to AlPO_4_ compared to silicates. This can be due, in our opinion, to a combination of at least two causes. First, layered aluminophosphates are more prone to hydrolysis during the necessary heating than silicates are. Second, since the connectivity between layers cannot likely occur through Al–O–Al and P–O–P bonds, this reduces the likelihood of a successful connection compared to silicates (consider, in comparison, that siliceous *fer* layers can condense into FER,^37^ or alternatively into CDO type zeolites,^38,39^ while a similar condensation to a new phase would be prevented by the Löwestein rule in HPM-3). As we show here, reaching a too high energy by thermal treatments may introduce 1D-stacking disorder, favored too by the second cause above, and promote hydrolysis, finally yielding almost amorphous materials. Thus, low thermal detemplation methods, such as ozonolysis, may be worth of pursuit when layered aluminophosphates fail to produce microporous AlPO_4_ phases.

## Conclusions

A new layered AlPO_4_ material, HPM-3, synthesized using 123TMI by the fluoride route is presented. It contains negatively charged Al(OP)_4_FAl(OP)_4_ pairs and Al(OP)_3_OH groups that balance the charge of the occluded cations. Interestingly, the layers in HPM-3 were found to be identical to those in MAPO molecular sieves with **JSN** topology. Therefore, HPM-3 can be a precursor to a metal-free AlPO_4_ analog with **JSN** topology. Attempts to produce the required topotactic condensation under different conditions generally failed and generally produced at most ill-crystallized **JSN** phase in a very limited range of conditions. Along the way we obtained a number of other phases including monoclinic and hexagonal **AFI** solids through disruptive transformations and a modification of HPM-3 itself with shorter interlayer spacings (HPM-3S) through a topotactic transition that does not reach, however, condensation. This topotactic transition is possible thanks to a low temperature sublimation of OSDA and may also result in transient phases with different interlayer spacings.

A structural inspection of the structure of HPM-3 suggests that the difficulty in obtaining an easy and perfect topotactic condensation is related to a misalignment of the terminal P = O and Al–OH groups that should serve as connection points. This affords random shifts of the layers upon the thermal treatments needed to remove the organics, resulting in low crystallinity materials with extreme 1-D stacking disorder. However, it is possible to get AlPO_4_-**JSN** with only moderate disorder by a low temperature (175 °C) treatment with O_3_, suggesting the random shifts are minimized under those conditions. The results presented here show that novel 2D layered structures and 3D zeolite structures, which may be very difficult to achieve by direct synthesis, can be obtained by careful postsynthetic treatments (physical, chemical, or both) of presynthesized layered materials. This may stimulate further research on the phase transformations of presynthesized layered materials.

## Supporting Information Available

Additional PXRD patterns, TG/DTA curves, ^31^P and ^13^C MAS NMR spectra, TPD profile, ATR-FTIR spectra, Rietveld refinement plots, FE-SEM, cRED and structural figures as well as tabulated synthesis results, cRED data, crystallographic data, bond length and angle data, and summary of phase transformations in air and N_2_ (PDF)

## References

[ref1] Baerlocher C.; McCusker L. B. Database of Zeolite Structures. http://www.iza-structure.org/databases/ (accessed October 10, 2024).

[ref2] Davis M. E.; Lobo R. F. Zeolite and molecular sieve synthesis. Chem. Mater. 1992, 4, 756–768. 10.1021/cm00022a005.

[ref3] Lee J. H.; Kim E. J.; López-Arbeloa F.; Hong S. B.; Camblor M. A. Microporous aluminophosphates synthesized with 1,2,3-trimethylimidazolium and fluoride. Dalton Trans. 2016, 45, 7616–7626. 10.1039/C6DT00734A. 27048542

[ref4] Rojas A.; Martínez-Morales E.; Zicovich-Wilson C. M.; Camblor M. A. Zeolite Synthesis in Fluoride Media: Structure Direction toward ITW by Small Methylimidazolium Cations. J. Am. Chem. Soc. 2012, 134, 2255–2263. 10.1021/ja209832y. 22239228

[ref5] Yu J.; Xu R. Insight into the construction of open-framework aluminophosphates. Chem. Soc. Rev. 2006, 35, 593–604. 10.1039/b505856m. 16791331

[ref6] Cichocka M. O.; Ångström J.; Wang B.; Zou X.; Smeets S. High-throughput continuous rotation electron diffraction data acquisition via software automation. J. Appl. Crystallogr. 2018, 51, 1652–1661. 10.1107/S1600576718015145. 30546290 PMC6276279

[ref7] Liu Z.; Song X.; Li J.; Li Y.; Yu J.; Xu R. |(C_4_NH_12_)_4_|[M_4_Al_12_P_16_O_64_] (M = Co, Zn): New Heteroatom-Containing Aluminophosphate Molecular Sieves with Two Intersecting 8-Ring Channels. Inorg. Chem. 2012, 51, 1969–1974. 10.1021/ic2022903. 22250971

[ref8] Schreyeck L.; Caullet P.; Mougenel J.-C.; Guth J.-L.; Marler B. A layered microporous aluminosilicate precursor of FER-type zeolite. J. Chem. Soc., Chem. Commun. 1995, 0, 2187–2188. 10.1039/c39950002187.

[ref9] Li J.; Gao Z. R.; Lin Q.-F.; Liu C.; Gao F.; Lin C.; Zhang S.; Deng H.; Mayoral A.; Fan W.; et al. A 3D extra-large-pore zeolite enabled by 1D-to-3D topotactic condensation of a chain silicate. Science 2023, 379, 283–287. 10.1126/science.ade1771. 36656929

[ref10] Morris R. E. Clicking zeolites together. Science 2023, 379, 236–237. 10.1126/science.adf3961. 36656940

[ref11] Yu H.; Villaescusa L. A.; Gao Z. R.; Camblor M. A. Stable Silica-Based Zeolites with Three-Dimensional Systems of Extra-Large Pores. Angew. Chem., Int. Ed. 2024, e202412170 10.1002/anie.202412170. 39142293

[ref12] Inagaki S.; Yokoi T.; Kubota Y.; Tatsumi T. Unique adsorption properties of organic–inorganic hybrid zeolite IEZ-1 with dimethylsilylene moieties. Chem. Commun. 2007, 5188–5190. 10.1039/b713466e. 18060137

[ref13] Gao Z. R.; Yu H.; Chen F.-J.; Mayoral A.; Niu Z.; Niu Z.; Li X.; Deng H.; Márquez-Álvarez C.; He H.; et al. Interchain-expanded extra-large-pore zeolites. Nature 2024, 628, 99–103. 10.1038/s41586-024-07194-6. 38538794

[ref14] Opanasenko M.; Shamzhy M.; Wang Y.; Yan W.; Nachtigall P.; Čejka J. Synthesis and Post-Synthesis Transformation of Germanosilicate Zeolites. Angew. Chem., Int. Ed. 2020, 59, 19380–19389. 10.1002/anie.202005776. 32510709

[ref15] Richardson J. W.; Smith J. V.; Pluth J. J. AlPO_4_-25: framework topology, topotactic transformation from AlPO_4_-21, and high-low displacive transition. J. Phys. Chem. 1990, 94, 3365–3367. 10.1021/j100372a002.

[ref16] Wheatley P. S.; Morris R. E. Calcination of a layered aluminofluorophosphate precursor to form the zeolitic AFO framework. J. Mater. Chem. 2006, 16, 1035–1037. 10.1039/b518265d.

[ref17] Guo P.; Afeworki M.; Cao G.; Yun Y.; Sun J.; Su J.; Wan W.; Zou X. Synthesis and Structure of a Layered Fluoroaluminophosphate and Its Transformation to a Three-Dimensional Zeotype Framework. Inorg. Chem. 2018, 57, 11753–11760. 10.1021/acs.inorgchem.8b01890. 30156401

[ref18] Momma K.; Izumi F. VESTA 3 for three-dimensional visualization of crystal, volumetric and morphology data. J. Appl. Crystallogr. 2011, 44, 1272–1276. 10.1107/S0021889811038970.

[ref19] Gale J. D.; Rohl A. L. The General Utility Lattice Program (GULP). Mol. Simul. 2003, 29, 291–341. 10.1080/0892702031000104887.

[ref20] Balestra S. R. G.; Rodríguez-Sánchez N.; Mena-Torres D.; Ruiz-Salvador A. R. Structural Features and Zeolite Stability: A Linearized Equation Approach. Cryst. Growth Des. 2024, 24, 938–946. 10.1021/acs.cgd.3c00893. PMC10853909 38344677

[ref21] Gale J. D.; Henson N. J. Derivation of interatomic potentials for microporous aluminophosphates from the structure and properties of berlinite. J. Chem. Soc., Faraday Trans. 1994, 90, 3175–3179. 10.1039/ft9949003175.

[ref22] Altomare A.; Cuocci C.; Giacovazzo C.; Moliterni A.; Rizzi R.; Corriero N.; Falcicchio A. EXPO2013: a kit of tools for phasing crystal structures from powder data. J. Appl. Crystallogr. 2013, 46, 1231–1235. 10.1107/S0021889813013113.

[ref23] TOPAS Version 4.2; Bruker AXS: Karlsruhe, Germany, 2009.

[ref24] Yu L.; Pang W.; Li L. Synthesis and structure of a novel microporous crystal AlPO_4_ CJ2. J. Solid State Chem. 1990, 87, 241–244. 10.1016/0022-4596(90)90089-G.

[ref25] Férey G.; Loiseau T.; Lacorre P.; Taulelle F. Oxyfluorinated Microporous Compounds. I. Crystal Structure of (NH_4_)_0.93_(H_3_O)_0.07_GaPO_4_(OH)_0.5_F_0.5_; Reexamination of the Structure of AlPO_4_-CJ2. J. Solid State Chem. 1993, 105, 179–190. 10.1006/jssc.1993.1206.

[ref26] Huo Q.; Xu R.; Li S.; Xu Y.; Ma Z.; Yue Y.; Li L. Synthesis, Characterization and Phase Transition of the 20-Membered Ring AlPO_4_: JDF-20. Proceedings from the Ninth International Zeolite Conference (Montreal 1992), 1993; Ballmoos R. v.; Higgins J. B.; Treacy M. M. J., Eds.; Butterworth-Heinemann: pp 279–286.

[ref27] Jones R. H.; Thomas J. M.; Chen J.; Xu R.; Huo Q.; Li S.; Ma Z.; Chippindale A. M. Structure of an Unusual Aluminium Phosphate ([Al_5_P_6_O_24_H]^2-^ 2[N(C_2_H_5_)_3_H]^+^ · 2H_2_O) JDF-20 with Large Elliptical Apertures. J. Solid State Chem. 1993, 102, 204–208. 10.1006/jssc.1993.1023.

[ref28] Müller D.; Jahn E.; Ladwig G.; Haubenreisser U. High-resolution solid-state ^27^Al and ^31^P NMR: correlation between chemical shift and mean Al-O-P angle in AlPO_4_ polymorphs. Chem. Phys. Lett. 1984, 109, 332–336. 10.1016/0009-2614(84)85596-7.

[ref29] Tuel A.; Lorentz C.; Gramlich V.; Baerlocher C. Synthesis, characterization and structure determination of two isotypes of a layered aluminophosphate with a new 2D network topology. J. Solid State Chem. 2005, 178, 2322–2331. 10.1016/j.jssc.2005.05.010.

[ref30] Taulelle F.; Pruski M.; Amoureux J. P.; Lang D.; Bailly A.; Huguenard C.; Haouas M.; Gérardin C.; Loiseau T.; Férey G. Isomerization of the Prenucleation Building Unit during Crystallization of AlPO_4_-CJ2: An MQMAS, CP-MQMAS, and HETCOR NMR Study. J. Am. Chem. Soc. 1999, 121, 12148–12153. 10.1021/ja991295n.

[ref31] Wan W.; Sun J.; Su J.; Hovmöller S.; Zou X. Three-dimensional rotation electron diffraction: software RED for automated data collection and data processing. J. Appl. Crystallogr. 2013, 46, 1863–1873. 10.1107/S0021889813027714. 24282334 PMC3831301

[ref32] Kabsch W. XDS. Acta Cryst. 2010, D66, 125–132. 10.1107/S0907444909047337. PMC2815665 20124692

[ref33] Sheldrick G. M. SHELXT – Integrated space-group and crystal-structure determination. Acta Crystallogr. 2015, A71, 3–8. 10.1107/S2053273314026370. PMC4283466 25537383

[ref34] Dolomanov O. V.; Bourhis L. J.; Gildea R. J.; Howard J. A. K.; Puschmann H. OLEX2: a complete structure solution, refinement and analysis program. J. Appl. Crystallogr. 2009, 42, 339–341. 10.1107/S0021889808042726.

[ref35] Toby B. H.; Von Dreele R. B. GSAS-II: the genesis of a modern open-source all purpose crystallography software package. J. Appl. Crystallogr. 2013, 46, 544–549. 10.1107/S0021889813003531.

[ref200] Cao X.; Wang P.; Wan R.; Guo C.; Tian S. Structural insight of fluorophosphate glasses through F/O ratio: Case study of Raman and NMR spectra. J. Non-Cryst. Solids 2024, 637, 123065 10.1016/j.jnoncrysol.2024.123065.

[ref201] Möncke D.; Eckert H. Review on the structural analysis of fluoride-phosphate and fluoro-phosphate glasses. J. Non-Cryst. Solids X 2019, 3, 100026 10.1016/j.nocx.2019.100026.

[ref36] Gao Z. R.; Márquez-Álvarez C.; Balestra S. R. G.; Yu H.; Villaescusa L. A.; Camblor M. A. Mechanism of the Low-Temperature Organic Removal from Imidazolium-Containing Zeolites by Ozone Treatment: Fluoride Retention in Double-4-Rings. Inorg. Chem. 2024, 63, 9953–9966. 10.1021/acs.inorgchem.4c01021. 38757795 PMC11134512

[ref37] Schreyeck L.; Caullet P.; Mougenel J. C.; Guth J. L.; Marler B. PREFER: a new layered (alumino) silicate precursor of FER-type zeolite. Microporous Materials 1996, 6, 259–271. 10.1016/0927-6513(96)00032-6.

[ref38] Dorset D. L.; Kennedy G. J. Crystal Structure of MCM-65: An Alternative Linkage of Ferrierite Layers. J. Phys. Chem. B 2004, 108, 15216–15222. 10.1021/jp040305q.

[ref39] Roth W. J.; Dorset D. L. The Role of Symmetry in Building up Zeolite Frameworks from Layered Zeolite Precursors Having Ferrierite and CAS Layers. Struct. Chem. 2010, 21, 385–390. 10.1007/s11224-009-9540-y.

