# Supplementary material for: Structure, and Phase Transformations of HPM-3: A Layered Precursor of the Neutral Aluminophosphate with JSN Topology
Source: Inorg Chem. 2024 Dec 9;63(51):24429–39. doi: 10.1021/acs.inorgchem.4c04881 (PMC11918533; doi:10.1021/acs.inorgchem.4c04881)
Supplement: Supplementary file 1 — ic4c04881_si_001.pdf [file ic4c04881_si_001.pdf]

Supporting Information

Structure, and Phase Transformations of HPM-3: A Layered Precursor of the Neutral Aluminophosphate with JSN Topology

Huajian Yu,[1] Eun Jeong Kim,[2] Zihao Rei Gao,[1,‡] Jeong Hwan Lee,[2,‖] Salvador R. G. Balestra,[1,3] Chao Ma,[4] Jian Li,[4] Carlos Márquez-Álvarez,[5] Bernd Marler,[6] Suk Bong Hong,[2,*] and Miguel A. Camblor[1,*]

1 Instituto de Ciencia de Materiales de Madrid (ICMM), CSIC, 28049 Madrid, Spain.

2 Center for Ordered Nanoporous Materials Synthesis, Division of Environmental Science and Engineering, POSTECH, Pohang 37673, Korea.

3 Departamento de Física Atómica, Molecular y Nuclear, Área de Física Teórica, Universidad de Sevilla, Seville 41012, Spain.

4 State Key Laboratory of Coordination Chemistry, School of Chemistry and Chemical Engineering, Nanjing University, Nanjing, Jiangsu 210023, China.

5 Instituto de Catálisis y Petroleoquímica (ICP), CSIC, 28049 Madrid, Spain.

6 Institute für Geologie, Mineralogie und Geophysik, Ruhr-Universität Bochum, 44780 Bochum, Germany

* *E-mail: sbhong@postech.ac.kr; macamblor@icmm.csic.es*

‡ Current address: Department of Chemical and Biomolecular Engineering and Institute for NanoBioTechnology, Johns Hopkins University, Baltimore, MD 21218, USA.

‖ Current address: Department of Chemical Engineering, The Pennsylvania State University, University Park, Pennsylvania 16802, USA.

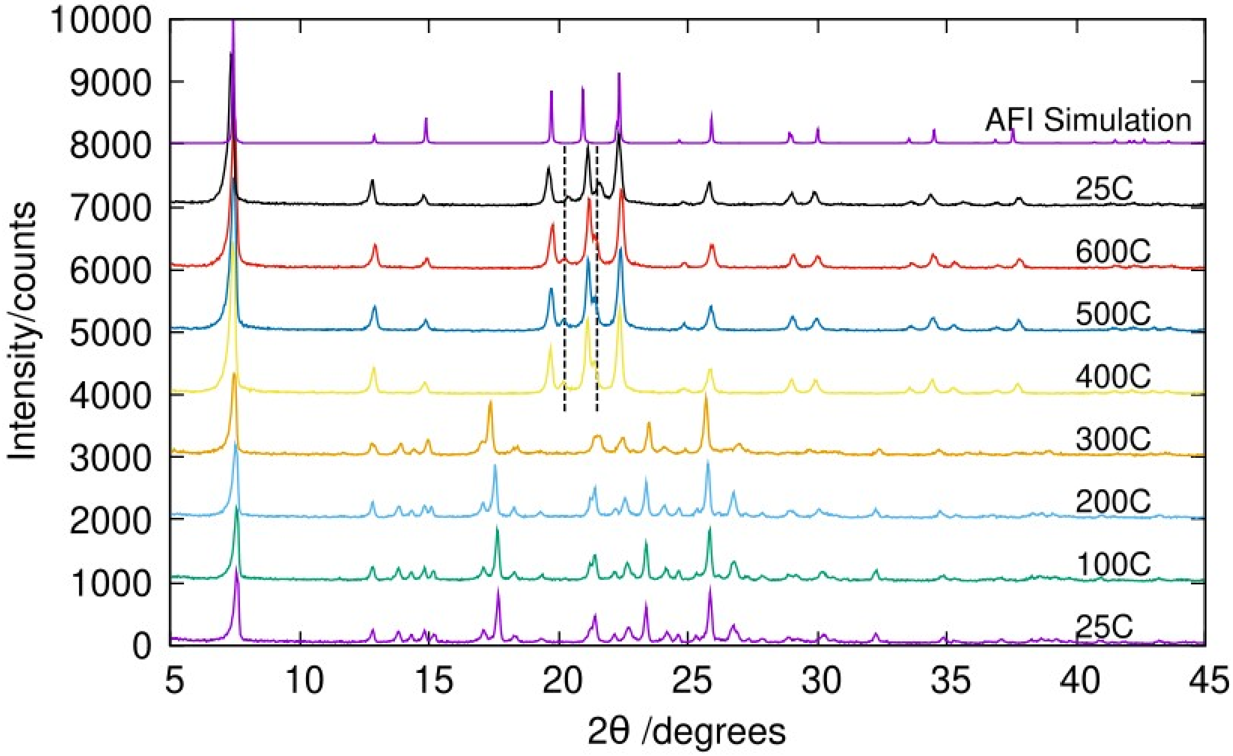

Figure S1. *In situ* PXRD patterns of HPM-3 upon heating in air at different temperatures. The top trace is a PXRD simulation for **AFI** using the data in the IZA-SC website.[1] Vertical traces mark a tridymite-like $AlPO_4$ impurity.

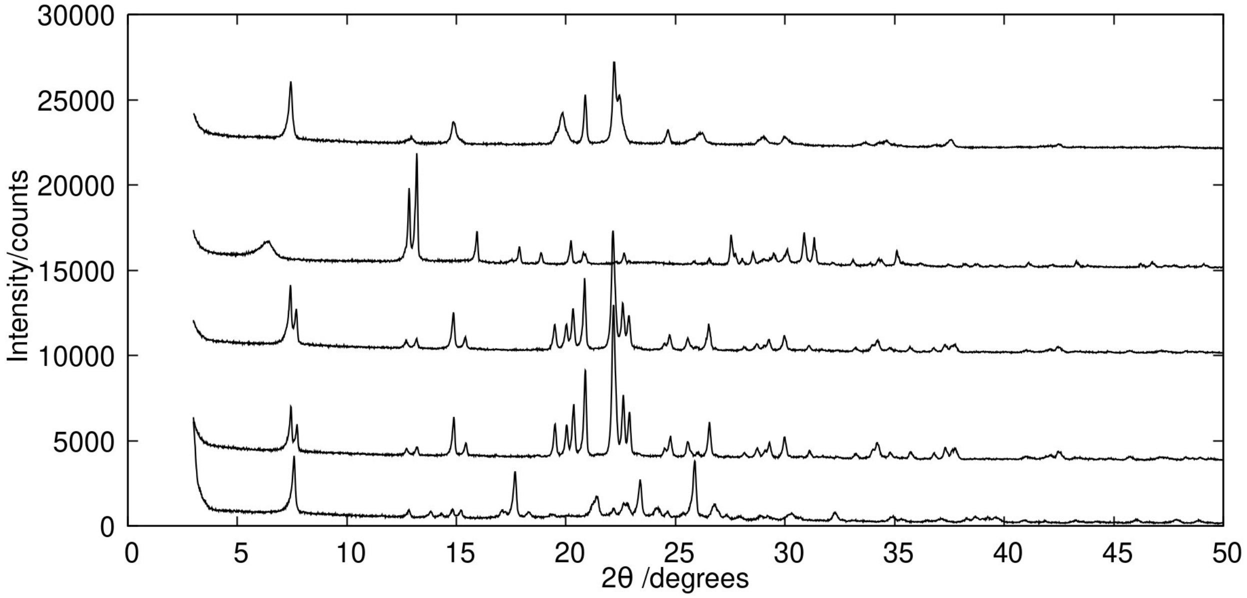

Figure S2. PXRD patterns of (from bottom to top) HPM-3 and its transformation products by digestion (180ºC, 3 h) in plain water (PST-27), water at pH=2.5 (PST-27), water at pH=10.5 ($AlPO_4$-CJ2) and a SAPO-HPM-3 in plain water ($AlPO_4$-5-like).

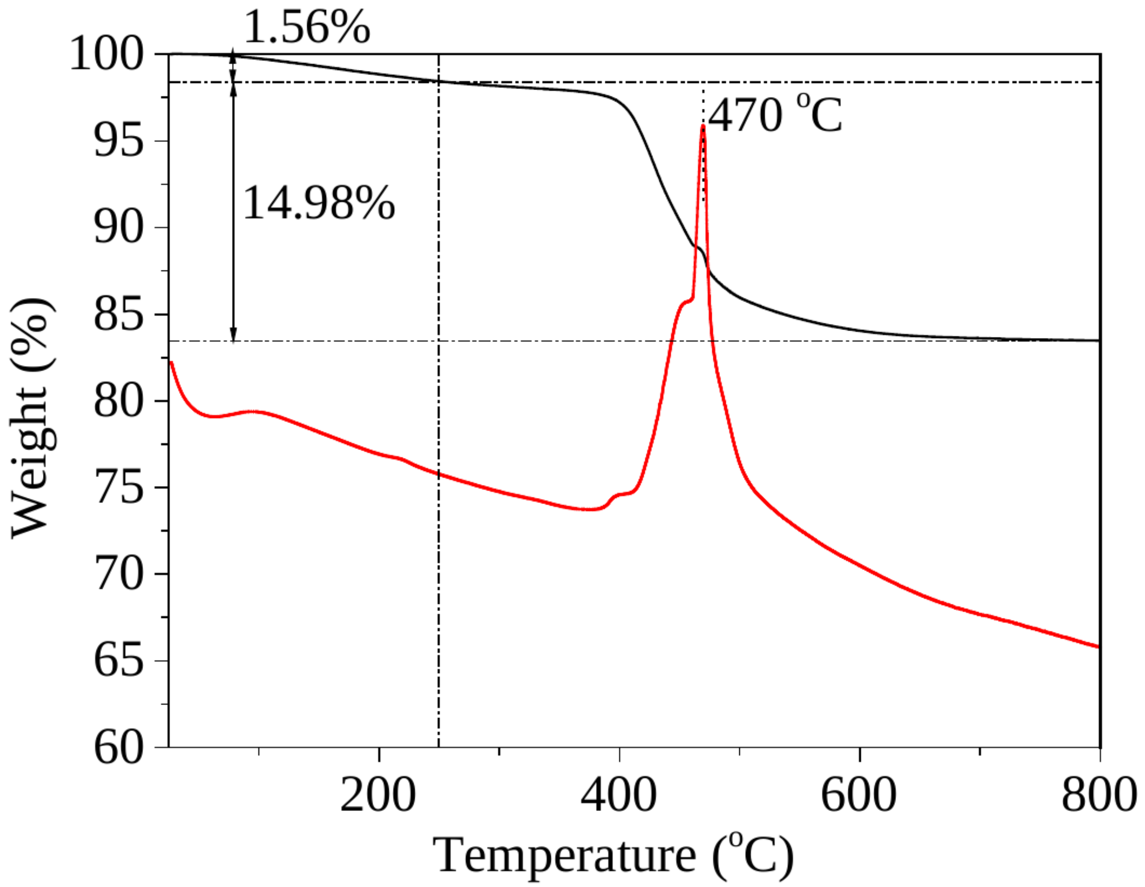

Figure S3. TG/DTA traces of the PST-27 sample obtained by digestion of HPM-3 at 180 ºC for 3 h.

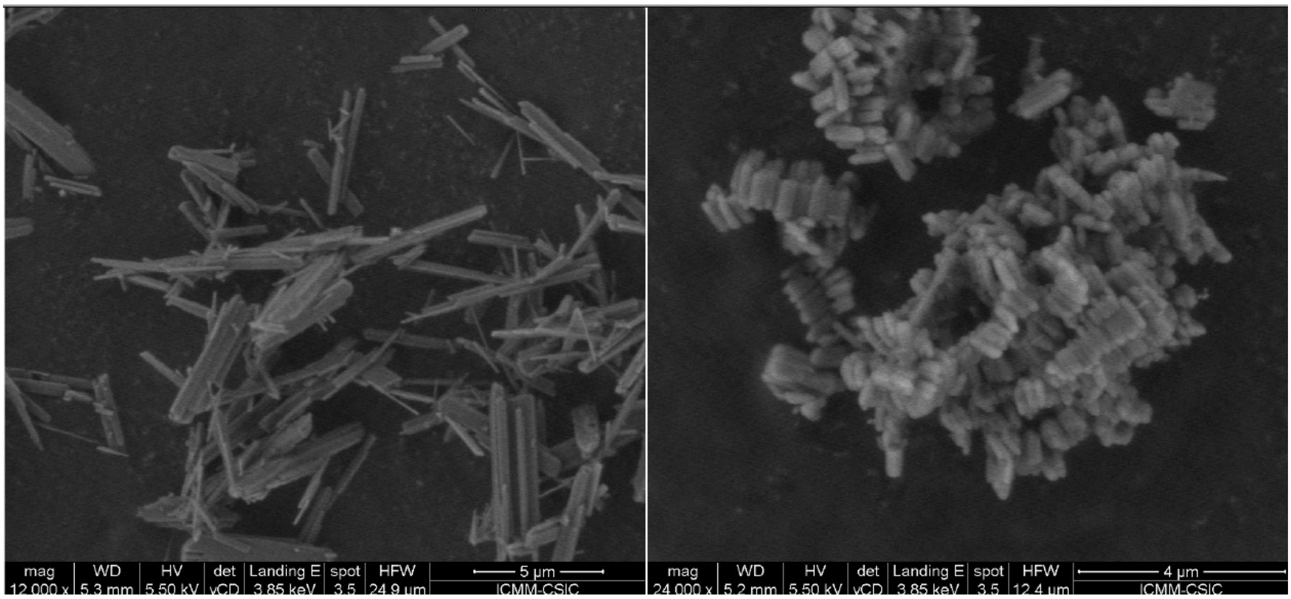

Figure S4. FE-SEM micrographs of as-synthesized HPM-3 (left) and the **AFI** phase obtained by calcination at 450 ºC (right).

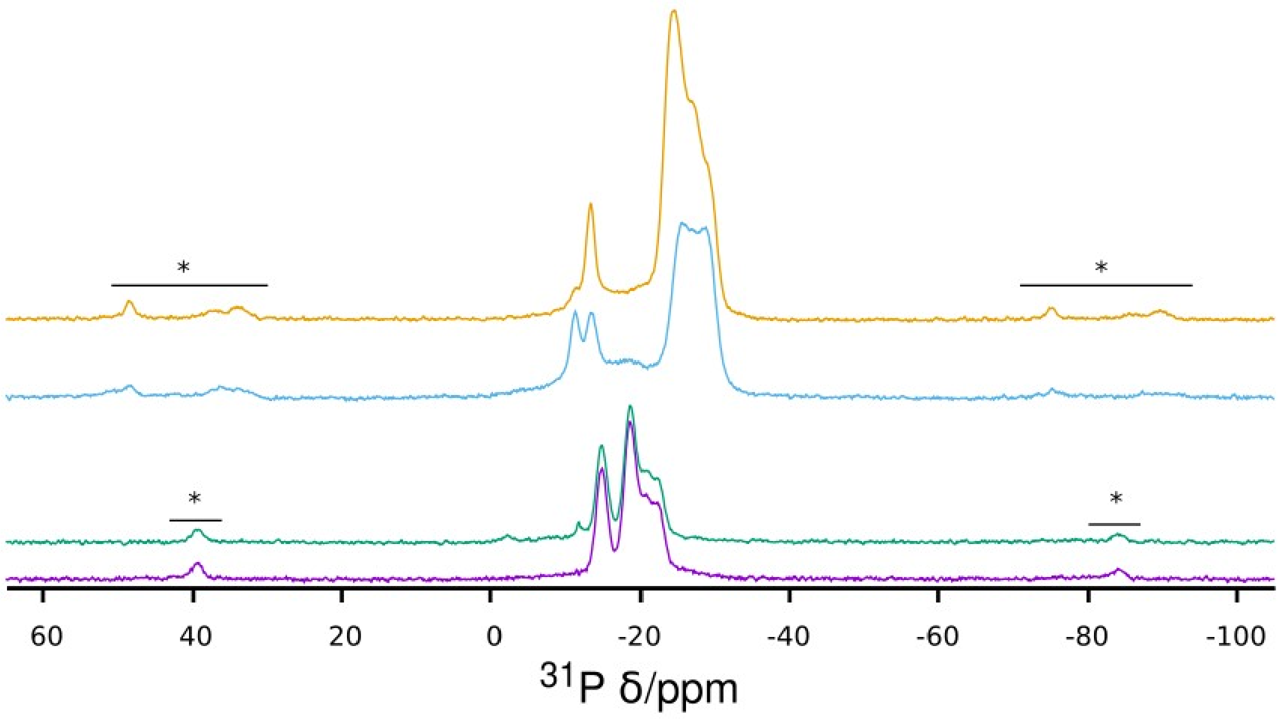

Figure S5. ^{31}P direct MAS NMR spectra of (from bottom to top) dried and undried HPM-3 and dried and undried HPM-3S materials. Regions of spinning side bands are marked with lines and asterisks.

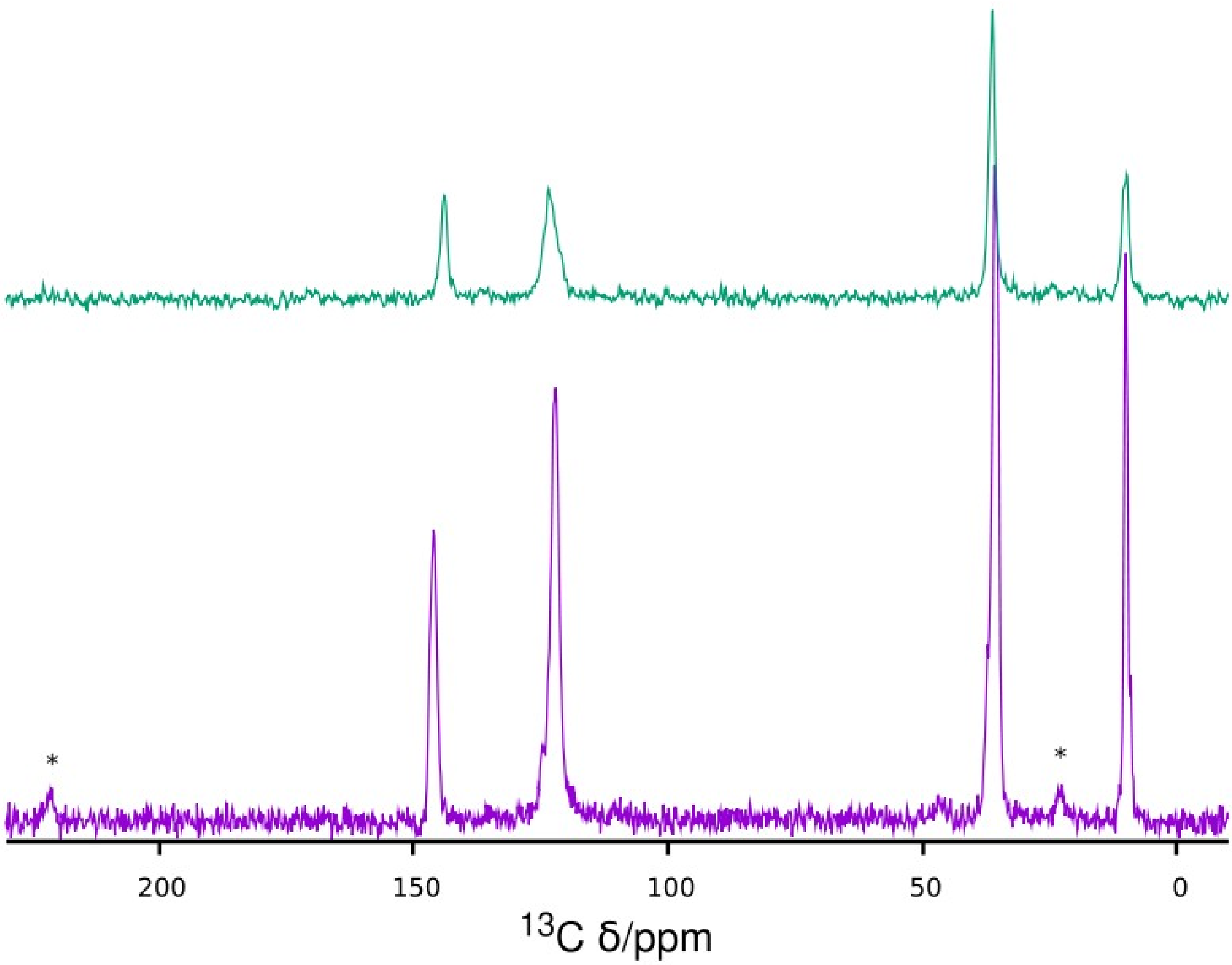

Figure S6. ^{1}H-^{13}C CPMAS NMR spectra of as-made HPM-3 (bottom) and HPM-3S (top), showing that the 123TMI cation is occluded intact and is not degraded by the treatment at 280 ºC for 48h. Spinning side bands are marked with asterisks.

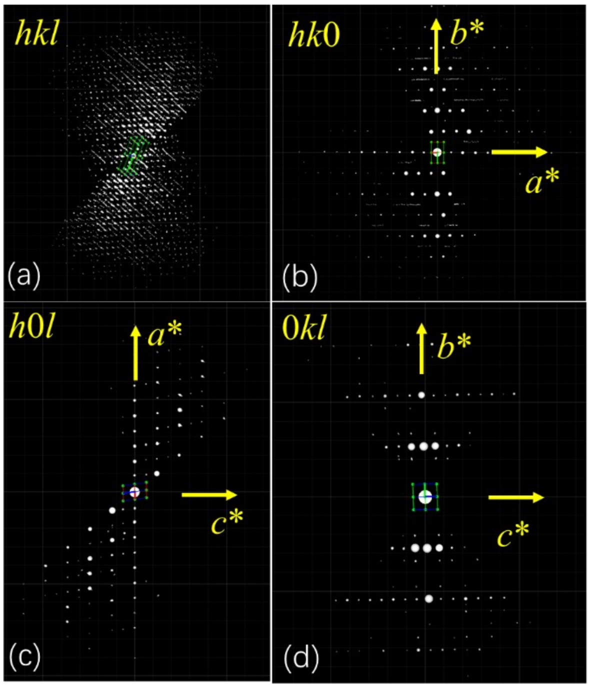

Figure S7. (a) Projection of 3D reciprocal space reconstructed from the typical cRED data of as-synthesized HPM-3 and the 2D slices of *hk*0 (b), *h*0*l* (c), and 0*kl* (d).

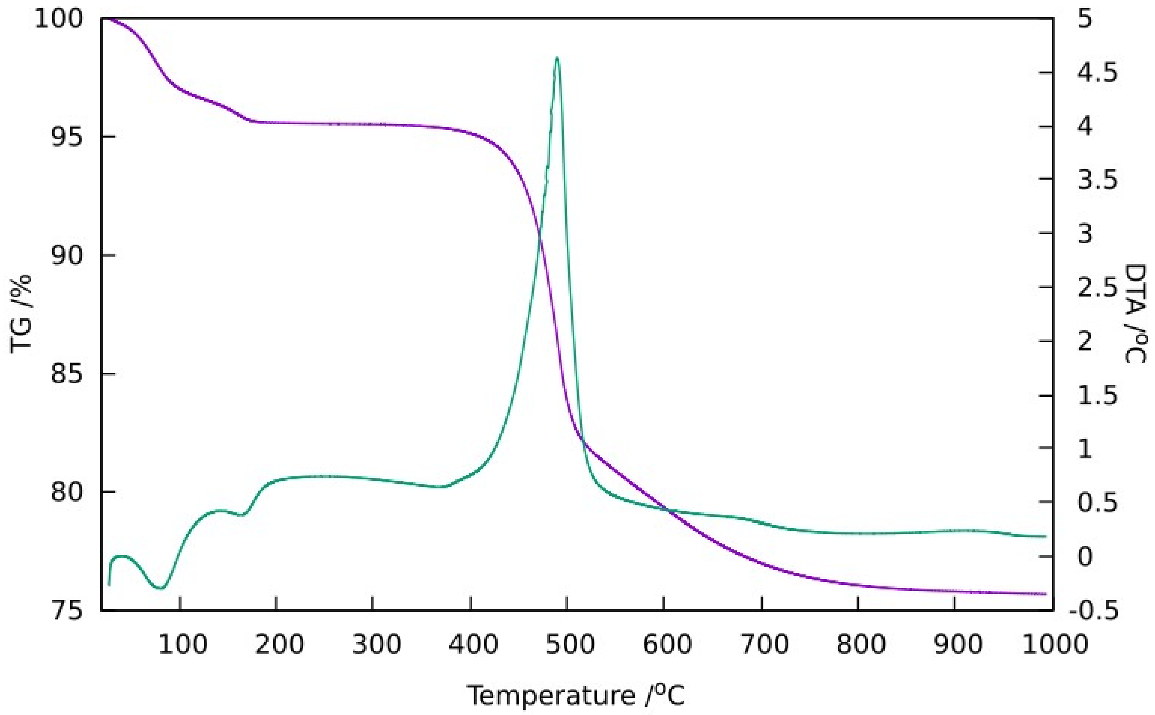

Figure S8. TG (purple) and DTA (green) curves of HPM-3S in air.

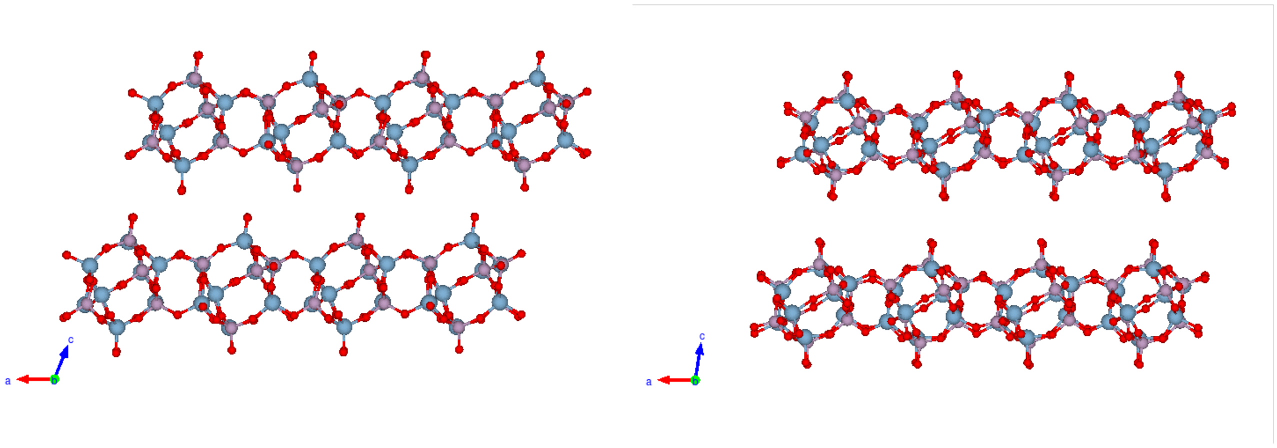

Figure S9. Comparison of the layered structures of HPM-3 (right) and HPM-3S (left) along *b*. Note that there is a significant lateral displacement of the layers along *a* in HPM-3S.

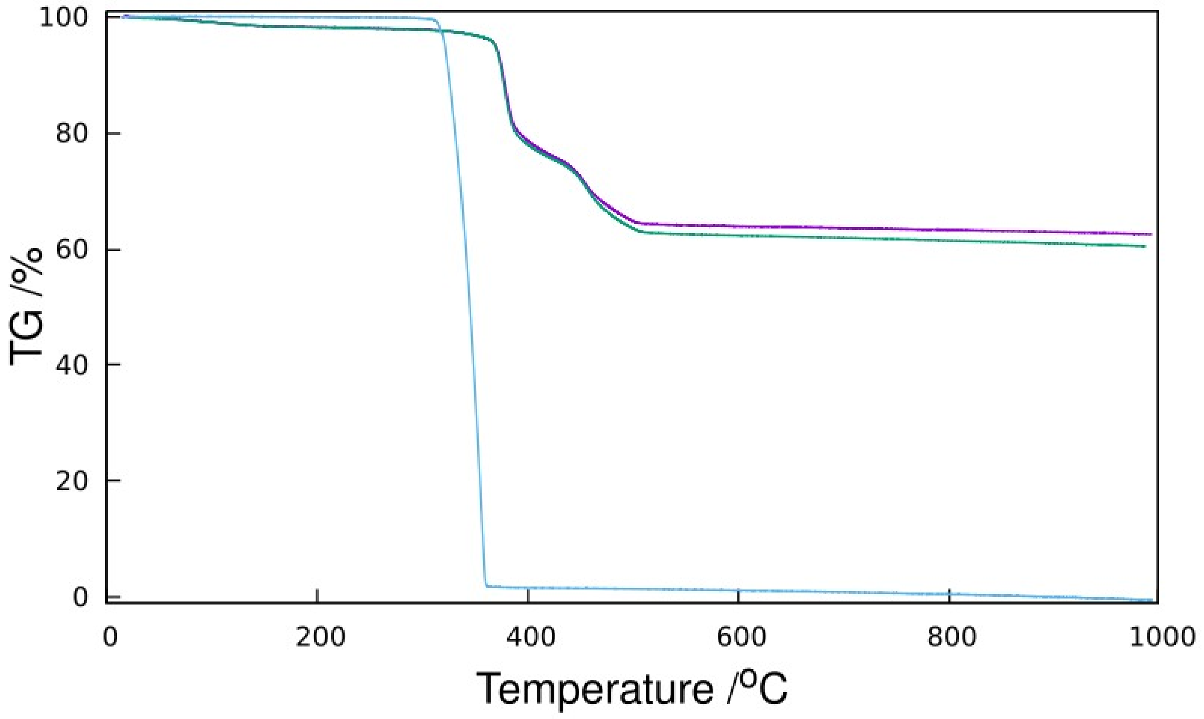

Figure S10. TG curves of HPM-3 under N_2 (purple) and Ar (green) and of 123TMI iodide under Ar (cyan).

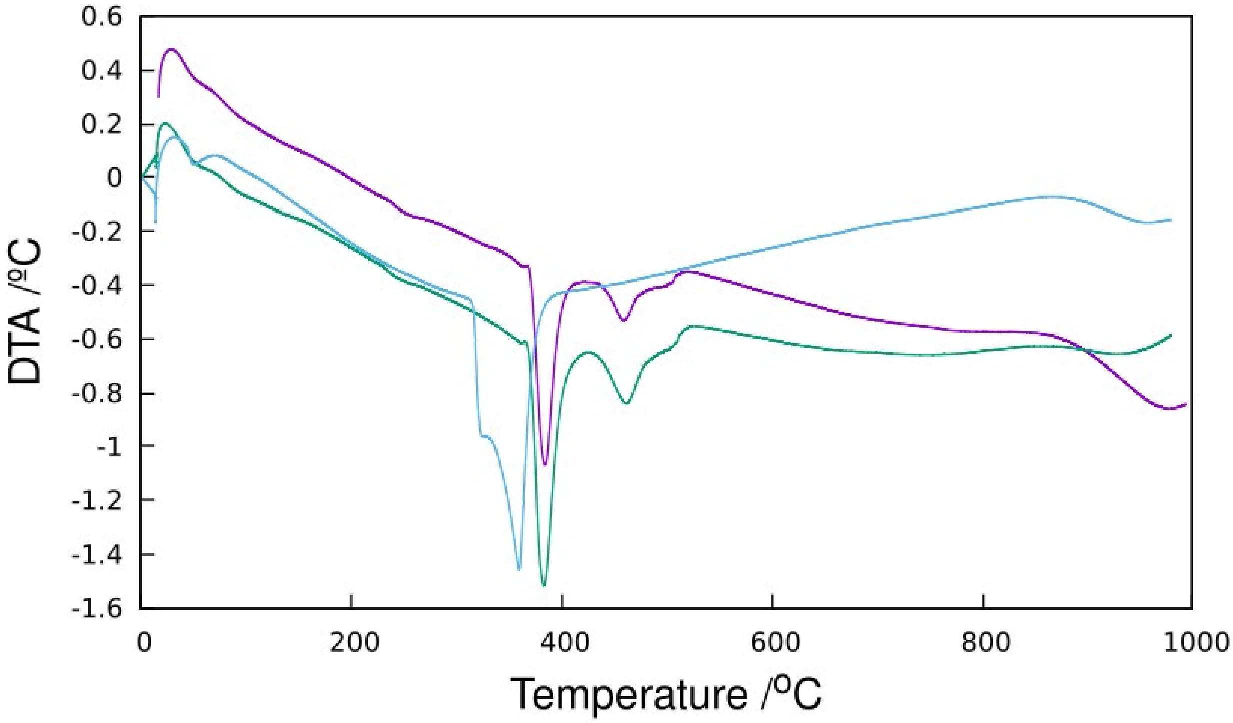

Figure S11. DTA traces of HPM-3 under N_2 (purple) and Ar (green) and of 123TMI iodide under Ar (cyan).

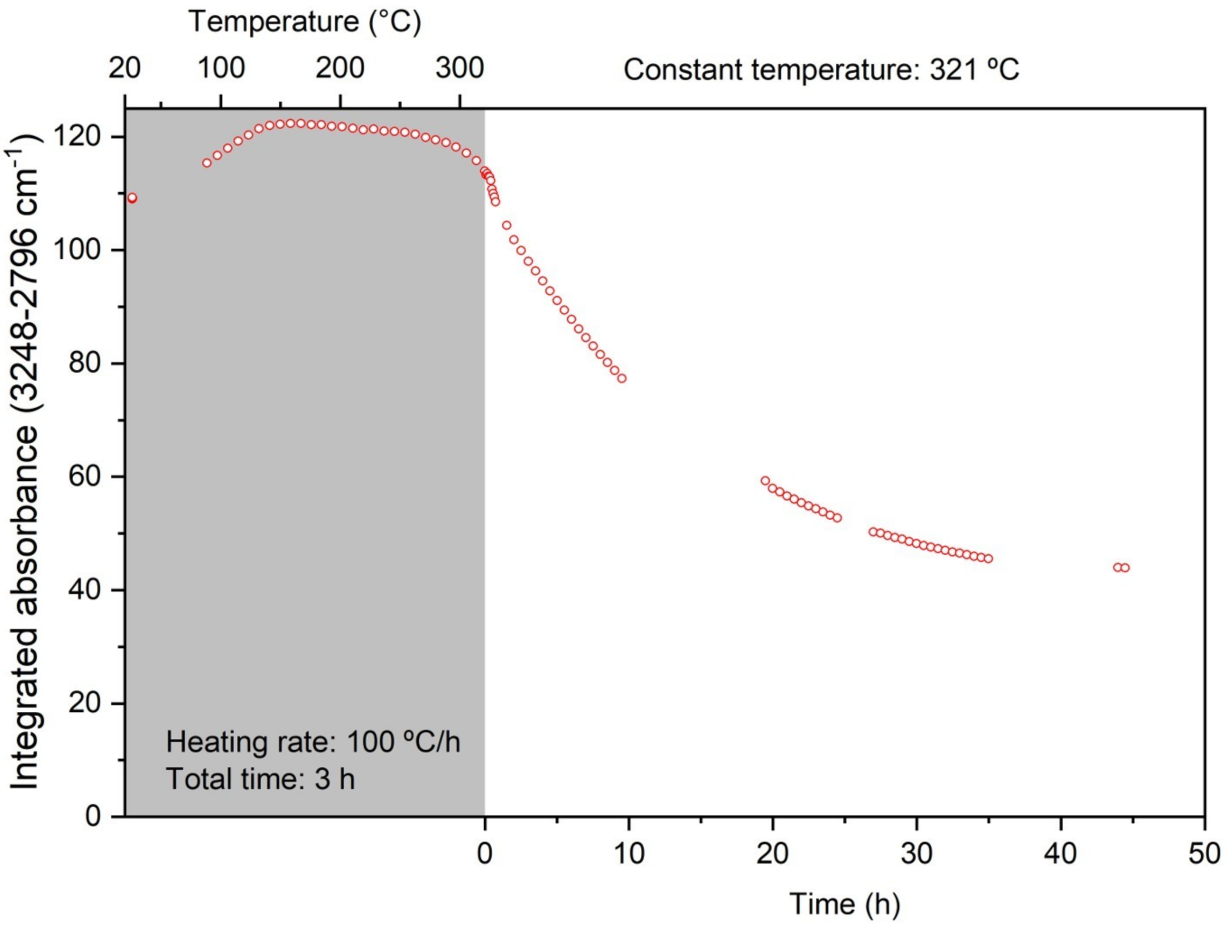

Figure S12. Temperature programmed desorption of HPM-3 under vacuum. The grey area corresponds to heating from room temperature to 321 ºC at a rate of 100 ºC/h, and its time scale has been expanded (around 8 times) compared to the white area of fixed temperature. The vertical axis is the integrated absorbance of the IR signals in the 3248-2976 cm^{-1}, covering the observed C-H stretching bands. Since the bands change during the TPD the intensity cannot be considered quantitative. However, it does reflect the slow removal of organics. The material after the TPD is HPM-3S.

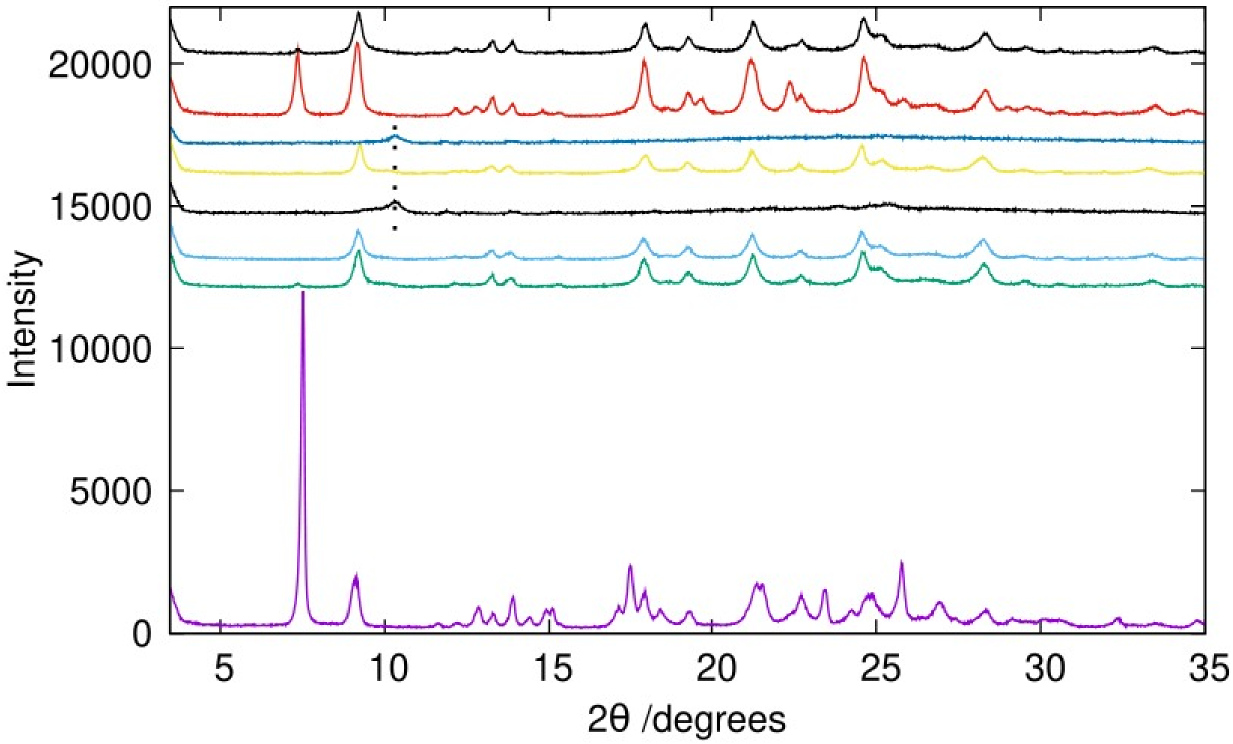

Figure S13. PXRD patterns of selected HPM-3 solids treated under N_2 flow at the following temperatures (ramps are always 6h, see Table S8), with phase identification between parentheses (from bottom to top): 6h at 320 ºC (HPM-3 + HPM-3S), 48h at 320 ºC (HPM-3S with traces of HPM-3), 96h at 320 ºC (HPM-3S), 48h at 320 ºC followed by 48h at 340 ºC (ill-crystallized **JSN**), 48h at 340 ºC (HPM-3S with some **JSN**), 48h at 320 ºC followed by 48h at 340 ºC and again 48h at 340 ºC (ill-crystallized **JSN**), 6h at 350 ºC (HPM-3S +**AFI**), 6h at 380 ºC (HPM-3S with some **AFI**). The dashed vertical line marks the d_{100} position for the **JSN** structure.

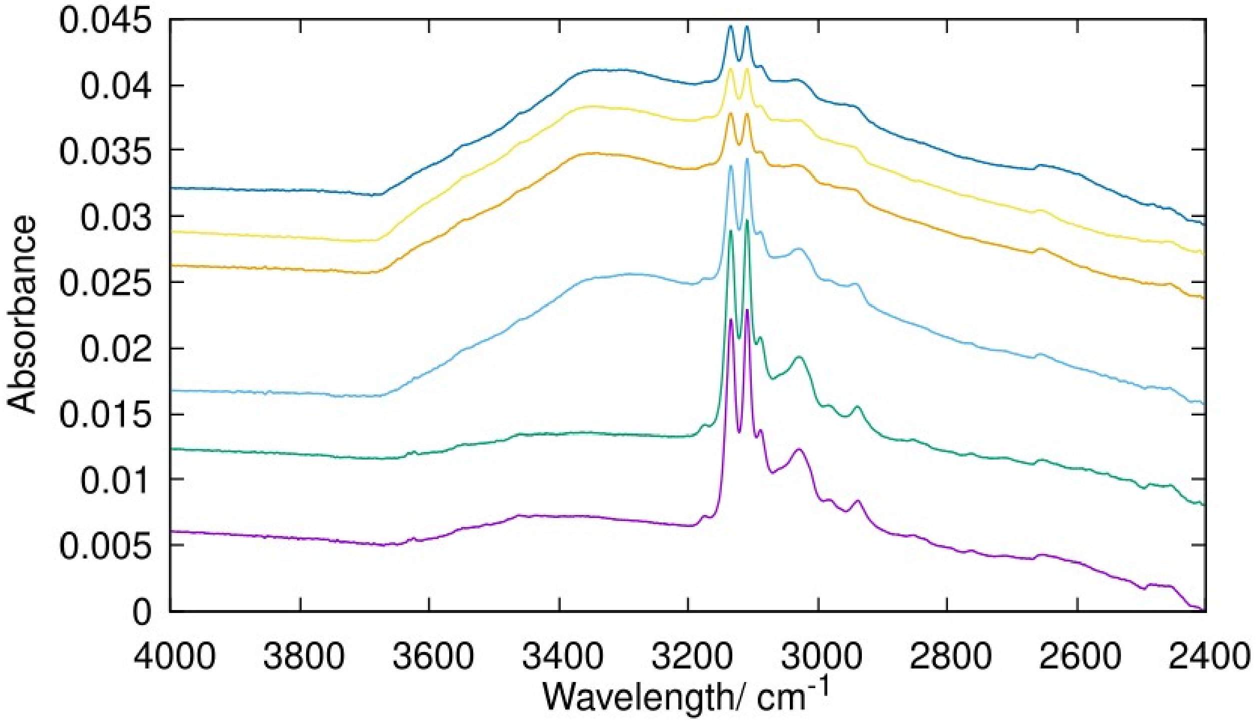

Figure S14. C-H stretching region of the ATR-FTIR spectra of (from bottom to top) as-made HPM-3 and its products after O_3 treatment at 80 ºC for 7 h, 100 ºC for 16 h, 150 º C for 8h, 150 ºC for 18 h, and 150 ºC for 35 h.

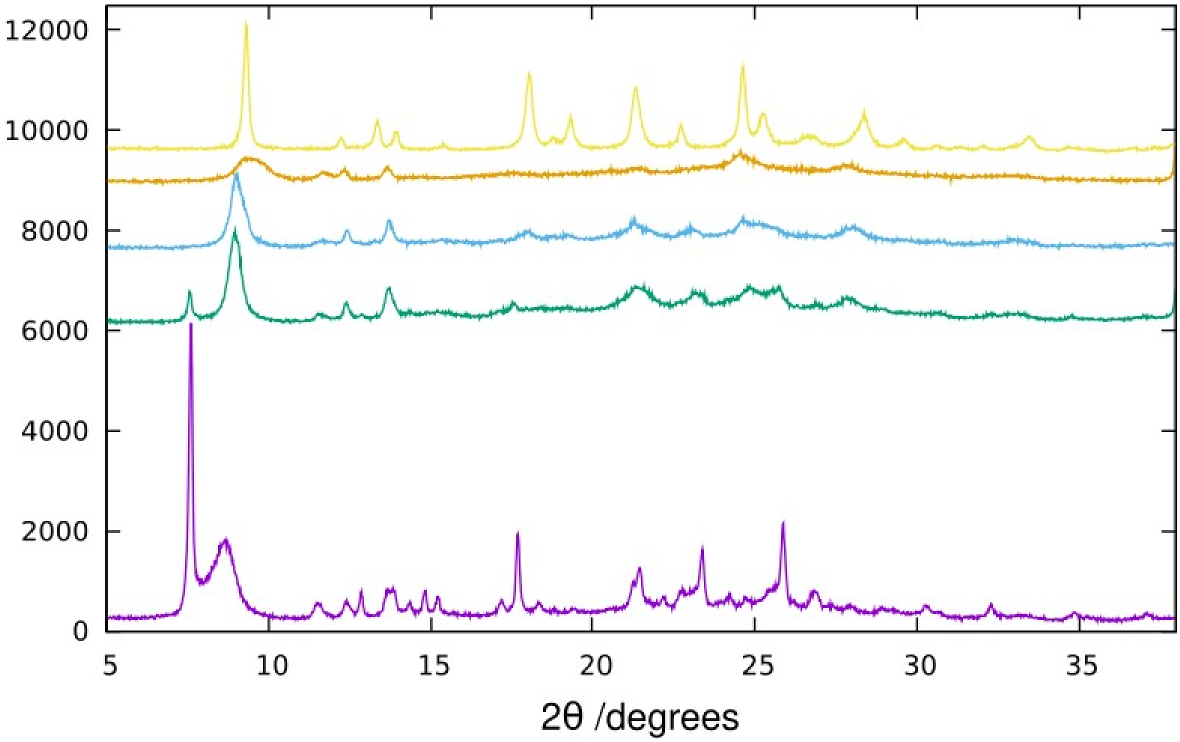

Figure S15. PXRD patterns of (from bottom to top): HPM-3 after ozonolysis at 150 ºC for 35 h, the same after calcination in N_2 at 250 ºC for 6 h, at 280 ºC for 48 h and at 340 ºC for 48h. The top pattern is HPM-3S (obtained by calcining HPM-3 in air at 280 ºC) for comparison.

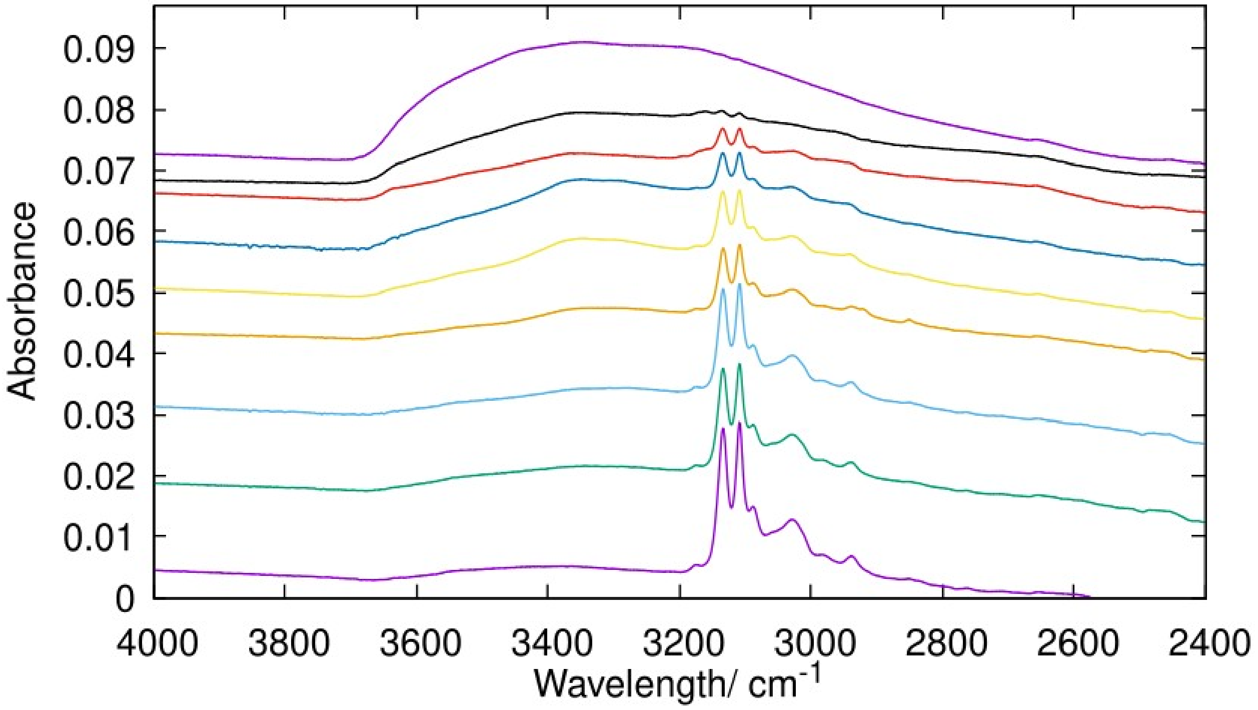

Figure S16. C-H stretching region of the ATR-FTIR spectra of HPM-3 (from bottom to top): as.made, and its products after O_3 treatment at 100 ºC for 6h, 100 ºC for 23 h, 150 ºC for 8h, 150 ºC for 24 h, 150 ºC for 96 h, 175 ºC for 5 h, 175 ºC for 9 h and 175 ºC for 24 h.

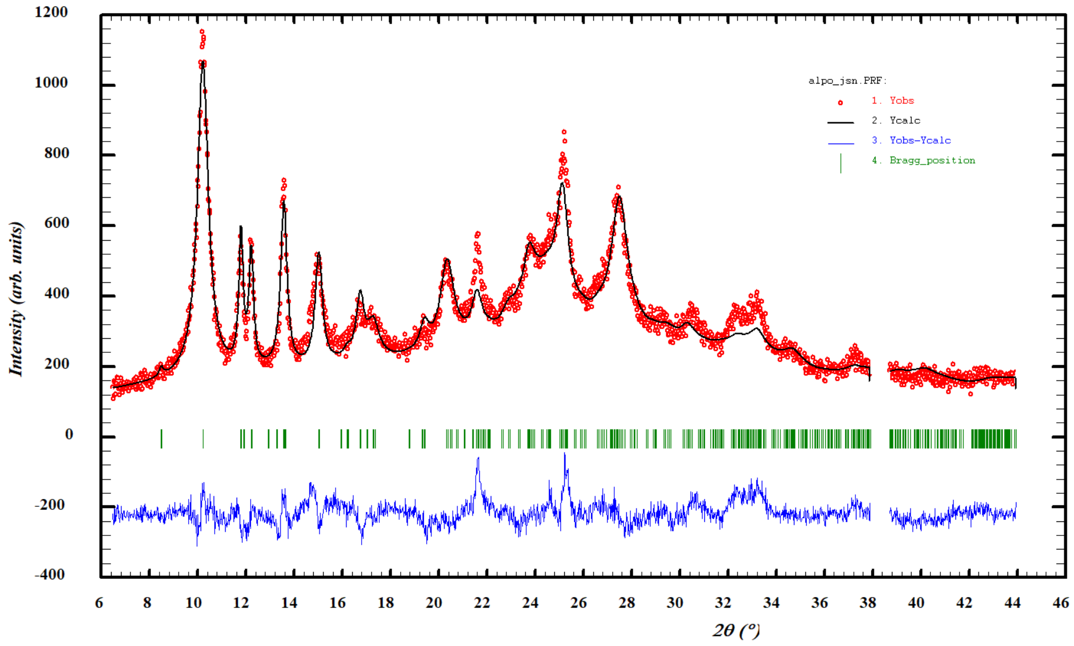

Figure S17. Plot of a Rietveld refinement of the phase obtained after O_3 treatment of HPM-3 at 175 °C for 25 h, followed by calcination in N_2 at 340 °C for 48 h (see Figure 10 in the main text). The starting structure model is an $AlPO_4$ with **JSN** structure type and the atomic coordinates were refined with restrains. Severe anisotropic broadening is observed, which could not be satisfactorily fit even with 6 additional parameters.

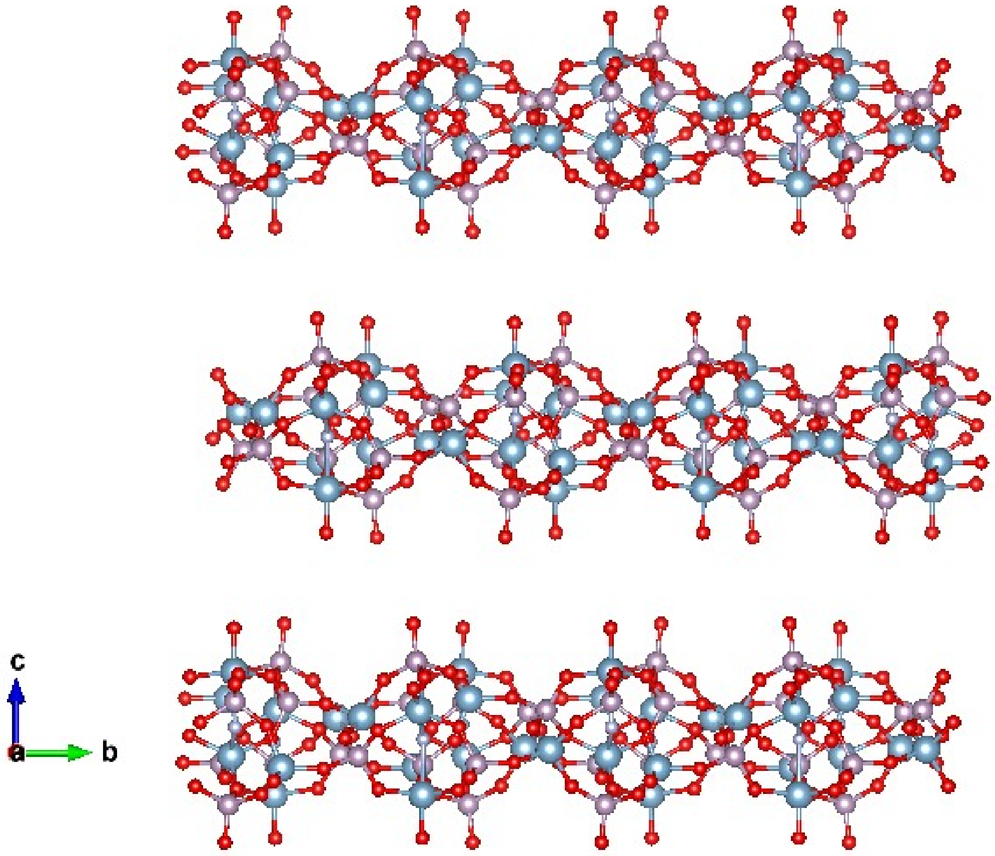

Figure S18. View of the structure of HPM-3 along *a*, showing the misalignment of Al-OH and P=O terminal groups.

Table S1. Synthesis Results for 2.0 123TMIOH : 1.0 Al_2O_3 : 1.0 P_2O_5 : 2.0 HF : w H_2O

w	Temp/ºC (time)	Product Crystal size (μm^3; FE-SEM)
5	150 (1, 6 d)	HPM-3 2x1x0.1 um3
5	150 (3 d)	HPM-3
10	150 (1, 4, 8 d)	HPM-3 10x2x0.1 um3
10	150 (1, 4, 7 d)	HPM-3
10	175 (2, 4, 8, 24 h)	HPM-3 10x2x0.2 um3

All synthesis were performed under rotation (60 rpm)

Table S2. cRED data indexing results for all crystals of long version HPM-3.

Data Set	SG	*a*/Å	*b*/Å	*c*/Å	*α*/°	*β*/°	*γ*/°	Volume/Å^3	Rotation angle/°	Total completeness
1	10	25.6	14.69	15.58	90	99.175	90	5797.672	115.072	67.4
2	10	24.9	15.08	16.22	90	102.38	90	5948.699	117.906	56.8
3	10	24.7	15.15	16.1	90	97.78	90	5974.077	120.484	69.1
4	10	24.6	15.04	16.01	90	96.995	90	5898.474	93.5145	57.9
5	10	24.4	15.04	16.15	90	98.741	90	5862.628	119.531	70.1
6	10	24.0	14.85	15.62	90	101.20	90	5467.602	38.3128	29.8
7	10	24.4	14.85	15.57	90	99.188	90	5587.509	47.2152	40.6
8	10	24.5	15	15.91	90	98.089	90	5805.291	89.088	56.5

Table S3. cRED data indexing results for all crystals of HPM-3S.

Data Set	SG	*a*/Å	*b*/Å	*c*/Å	*α*/°	*β*/°	*γ*/°	Volume/Å^3	Rotation angle/°	Total completeness
1	14	14.9	14.35	22.47	90	108.64	90	4555.464	57.1936	46.9
2	14	15.3	14.19	24.15	90	111.16	90	4911.991	49.88	35.4
3	14	15.5	14.36	24.11	90	112.42	90	4989.577	70.8111	38.2
4	14	15.4	14.56	24.82	90	115.30	90	5031.270	87.1192	49
5	14	15.2	14.59	23.73	90	111.67	90	4897.030	80.388	49.3
6	14	15.5	14.92	21.02	90	110.78	90	4562.251	9.0539	9.9

Table S4. cRED: Experimental parameters, crystallographic data and structure refinement details of HPM-3 and HPM-3S.

**Experimental parameters and crystallographic data**		
Identification code	HPM3	HPM-3S
Number of datasets	8	6
Wavelength	0.0251 Å	0.0251 Å
Spot size	3	3
Rotation range per image (°)	0.23	0.23
Exposure time per image (s)	0.5	0.5
Program for data procession	*XDS*	*XDS*
Program for structure solution	ShelxT	ShelxT
Crystal system	Monoclinic	Triclinic
Unit cell dimensions	*a* = 16.057(3)Å<br>*b* = 15.064(3)Å<br>*c* = 24.767(5)Å<br>*β* = 99.51(3)°	*a* = 12.410(3)<br>*b* = 14.370(3)Å<br>*c* = 15.612(3)Å<br>α= 91.65(3) °<br>β = 111.15(3)°<br>γ= 104.76(3) °
Possible space group	$P2_1/n$	*P*1
Resolution	0.81	0.81
Completeness	98.1%	75.3%
R_{int}	62.52%	31.32%
No. of reflections	104352	32175
No. of unique reflections	10905	12513
**structure refinement against cRED data**		
Formula	$C_{96}H_{176}N_{32}O_{136}F_8Al_{32}P_{32}$	$Al_{16}P_{16}O_{68}$
Crystal system	Monoclinic	Triclinic
Space group	$P2_1/n$	*P*1
Unit cell dimensions	*a* = 16.057(3)Å<br>*b* = 15.064(3)Å<br>*c* = 24.767(5)Å<br>*β* = 99.51(3)°	a = 12.410(3)<br>b = 14.370(3)Å<br>c = 15.612(3)Å<br>α= 91.65(3) °<br>β = 111.15(3)°<br>γ= 104.76(3) °
Dataset (*h*,*k l*)	-19~18, -18~18, -30~30	-13~12, -16~16, -17~17
Tot., Uniq. Data, Rint	104352, 10905, 62.52%	32175, 12513,31.32%
Observed Data [Fo > 4sig(Fo)]	1610	2210
$N_{reflections}$, $N_{parameters}$, $N_{restraints}$	104352, 326, 44	32175,401,2159
R_1, wR_2, *Gof*	0.3168, 0.69, 0.998	0.4781, 0.8134, 1.243
I/σ	1.9	2.6

ρ_{min}, ρ_{max} (e^- /Å^3)	-0.3/0.2	-0.3/0.3

Table S5. Crystallographic data for the Rietveld refinement for HPM-3.

Identification code	HPM-3
Empirical formula	$(AlPO_4)_{32}(OH)_8F_8(C_6N_2)_{16}$
Wavelength	0.82548 Å
Radiation	Synchrotron Radiation
Crystal system	Monoclinic
Space group	$P\,2_1/n$
Unit cell dimensions	a = 15.4052(7) Å<br>b = 14.4143(4) Å<br>c = 23.4905(11) Å<br>β = 98.9010(31)°
Volume	5153.37(19) Å^3
2θ range for data refinement	2.99° < 2θ < 35°
Number of parameters	271
Number of reflections	2086
Number of data points	3202
Number of restrains	32 for P-O, 32 for Al-O and 108 for O-P-O, O-Al-O and F-Al-O
Refinement method	Rietveld refinement
R_{wp}/GOF/*reduced chi**2*	0.06739/1.41/1.99

Table S6. Bond angles in HPM-3.

Bond angles(°)	Min	Max
O-P-O	102.8(9)	117.6(6)
O_{eq}-Al-O_{eq} (pentacoordinated)	112.4(8)	128.1(7)
O_{ap}-Al-O_{eq} (pentacoordinated)	90.0(9)	98.5(7)
O-Al-O (tetracoordinated)	103.6(0)	118.0(2)
F-Al-O	78.6(2)	91.3(2)

O_{eq} and O_{ap} are equatorial and apical O in the $Al(O)_4F$ trigonal bipyramid, respectively.

Table S7. Bond lengths in HPM-3.

atoms	length (Å)	atoms:	length (Å)
P1 - O6	1.535	Al1 - O6	1.813
P1 - O12	1.481	Al1 - O19	1.749
P1 - O16	1.538	Al1 - O30	1.797
P1 - O31	1.600	Al1 - O34	1.810
P2 - O1	1.539	Al2 - O4	1.775
P2 - O10	1.447	Al2 - O18	1.774
P2 - O17	1.494	Al2 - O24	1.763
P2 - O34	1.521	Al2 - O31	1.817
P3 - O3	1.557	Al3 - O7	1.810
P3 - O13	1.500	Al3 - O9	1.729
P3 - O15	1.508	Al3 - O13	1.774
P3 - O33	1.482	Al3 - O25	1.757
P4 - O9	1.526	Al4 - O5	1.770
P4 - O22	1.531	Al4 - O17	1.780
P4 - O23	1.536	Al4 - O32	1.757
P4 - O24	1.490	Al4 - O33	1.746
P5 - O18	1.557	Al5 - O3	1.780
P5 - O19	1.506	Al5 - O22	1.880
P5 - O20	1.547	Al5 - O28	1.749
P5 - O21	1.570	Al5 - O29	1.788
P6 - O4	1.484	Al6 - O1	1.774
P6 - O26	1.516	Al6 - O11	1.703
P6 - O27	1.529	Al6 - O16	1.842
P6 - O30	1.522	Al6 - O21	1.821
P7 - O5	1.448	Al7 - O2	1.761
P7 - O7	1.597	Al7 - O8	1.794
P7 - O8	1.556	Al7 - O20	1.782
P7 - O29	1.489	Al7 - O23	1.745
P8 - O2	1.532	Al8 - O10	1.781
P8 - O14	1.549	Al8 - O12	1.791
P8 - O25	1.544	Al8 - O15	1.687
P8 - O32	1.485	Al8 - O26	1.878

Table S8. Summary of Phase Transformations Undergone by HPM-3 upon Thermal Treatment in Air

Calcination procedure[a]	Product
deep bed, 450-2 h-6 h	**AFI**
deep bed, 500-2 h-6 h	**AFI**
shallow bed, 450-2 h-6 h	almost amorphous
shallow bed, 450-215 min - 4 h	almost amorphous
shallow bed, 500-2 h-6 h	amorphous
shallow bed, 500-24 min – 4 h	**AFI** + amorphous
shallow bed, 600-2 h-6 h	amorphous
shallow bed, 450-215 min-4 h	almost amorphous
SARC,[b] 200 ºC, 24 h	PST-27
SARC,[b] 190 ºC, 24 h	PST-27
SARC,[c] 190 ºC, 24 h	HPM-3
shallow bed, 190-3 h-168 h	HPM-3
shallow bed, 260-3 h-48 h	HPM-3S + HPM-3
shallow bed, 280-3 h-48 h	HPM-3S
shallow bed, 300-3 h-48 h	HPM-3S
shallow bed, 320-3 h-48 h	ill-crystallized **JSN**
deep bed, 280-3 h-6 h	HPM-3S + HPM-3

[a]*T*-t1-t2: treatment to *T* ºC with t1 ramp and t2 plateau. Deep bed and shallow bed calcinations relate to the thickness of the layer of solid to be calcined, based on the different amount of solid relative to the crucible volume. [b]Steam assisted recrystallization with 2 g water per 80mg HPM-3. [c3]Steam assisted recrystallization with 50 mg water per 80 mg HPM-3.

Table S9. Experiments under N_2[a]

Calcination Procedure	Product
6h 48h 320	HPM-3S
6h 48h 340	**JSN**(IC)+(HPM-3S)
6h 48h 340	**JSN**(IC)
6h 48h 320	HPM-3S
6h 48h 340	HPM-3S+**JSN**(IC)
6h 48h 340	**JSN**(IC)+HPM-3S(IC)
6h 48h 340	**JSN**(IC)
6h 48h 340	**JSN**(IC)
6h 6h 250	HPM-3
3h 48h 280	HPM-3+HPM-3S
6h 6h 320	HPM-3+HPM-3S
6h 96h 320	HPM-3S
6h 48h 340	HPM-3S+**JSN**(IC)
6h 48h 340 (large amount)	**JSN**(IC)
6h 6h 350	HPM-3S+**AFI**
6h 6h 380	HPM-3S+(**AFI**)
6h 48h 380	**AFI**+**JSN**(IC)
6h 6h 400	**AFI**+**JSN**(IC)
25min 4h 500	**AFI**
O_3-150-35h, 6h 6h 250	HPM-3T+HPM-3
6h 6h 250	HPM-3T+HPM-3
O_3-150-35h, 3h 48h 280	HPM-3T
6h 48h 340	HPM-3T2
6h 48h 340	**JSN**(IC)
O_3-175-24h, 6h 48h 340	**JSN**

[a] The treatments in each shaded block are consecutive (additional treatments of the sample already treated in the row above). For the unshaded block the treatments are independent. The amount of sample used in the second block is larger than the amount used in the others. "Transient" HPM-3T and HPM-3T2 refer to phases whose first diffraction peak is between those of HPM-3 and HPM-3S or HPM-3S and **JSN**, respectively.

References

1 Ch. Baerlocher and L.B. McCusker, Database of Zeolite Structures: http://www.iza-structure.org/databases/, accessed on October 2, 2024.
